# Enhancing PFAS data integrity: An LLM-based FAIR+Environmental principle for improved evaluation of environmental contaminants and related constituent databases

**DOI:** 10.1016/j.hazadv.2026.101103

**Published:** 2026-03-09

**Authors:** Jialin Dong, Sean D. Young, Zixin Hu, Christopher I Olivares

**Affiliations:** a Department of Civil and Environmental Engineering, University of California, Irvine, CA, USA; b Department of Informatics, University of California, Irvine, CA, USA; c Department of Emergency Medicine, University of California, Irvine, CA, USA

**Keywords:** PFAS, Water, Soil, AI, FAIR, Data

## Abstract

Per- and polyfluoroalkyl substances (PFAS) are persistent, bioaccumulative contaminants of emerging concern, yet data sharing of their environmental occurrence and monitoring remains fragmented. We propose a “FAIR+Environmental” framework that extends the Findable, Accessible, Interoperable, Reusable (FAIR) principles to environmental-specific matrices, assessed by a semi-automated Large Language Model (LLM) pipeline that uses rule-based scoring, few-shot prompting, and Chain-of-Thought (CoT) reasoning. We applied the framework to >100 U.S. PFAS datasets across groundwater, surface water, drinking water, and soil matrices. Few-shot CoT LLMs streamlined FAIR evaluations, reducing the manual effort required for expert assessments. Among environmental matrices, surface water datasets achieved the highest FAIR-Score (53.6 %), followed by drinking water (52.6 %), soil (49.3 %), and groundwater (45.2 %). Multi-state datasets consistently outperformed single-state datasets, particularly in Interoperability and Reusability criteria. Compared to geoscience databases, PFAS environmental datasets lag in FAIR adherence, highlighting the urgent need for centralized, standardized, and FAIR-compliant data management in the field. PFOA occurrence indicated overall PFAS pollution hotspots because of its frequent detection. PFOA contamination was most severe in soil and groundwater. Surface water and drinking water showed lower concentrations but remain critical public health exposures, with ~34 % of drinking water samples exceeding the 4 ng/L maximum contaminant level. Temporal trends indicated little significant change in PFOA concentrations across most states.

## 1. Introduction

Per- and polyfluoroalkyl substances (PFAS) are a class of over 15,000 synthetic, highly fluorinated chemicals that have been extensively used since the 1940s in products such as firefighting foams, metal plating, textiles, food packaging, and household goods (Scientists, 2024; Dong et al., 2024). Due to their exceptional chemical stability and resistance to degradation, PFAS are often called “forever chemicals” (Glüge et al., 2020). They have been widely detected in groundwater, surface water, soil, precipitation, and in the blood of nearly the entire U.S. population (Sonnenberg et al., 2023). In response to growing public and regulatory concerns, local and national agencies such as the U.S. Environmental Protection Agency (EPA) and the U.S. Geological Survey (USGS) have launched comprehensive PFAS monitoring initiatives at both federal and state levels for over a decade (Smalling et al., 2023; Hu et al., 2016). Individual databases have limited geographic coverage, different target analyte lists and methods, limiting evaluation across datasets. There is a large need to integrate the patchwork of individual datasets across all U. S. states and environmental media, to overcome challenges for large-scale environmental and public health assessment (Liddie et al., 2025; Dong et al., 2025).

Sampling campaigns differ regarding which PFAS compounds are prioritized, how frequently samples are collected, and what conditions trigger investigations. Compounding these scientific and regulatory inconsistencies are significant disparities in data management practices. Commercial testing laboratories and regulators use different standards for data formatting, metadata documentation, and submission protocols, making it difficult to harmonize datasets. Additional challenges in data quality include incomplete entries, a growing list of PFAS analytes, increasingly lower detection limits, and insufficient quality assurance/quality control (QA/QC) measures (e.g. missing field blanks) (Roll and Halden, 2016). Together, these limitations hinder effective data reuse and reduce the potential for integrated analysis to inform policy, environmental modeling, and risk assessment on broader scales.

Most of PFAS investigations tend to focus on one type of environmental media, such as groundwater, surface water, drinking water, or soil, rather than conducting integrated assessments across multiple matrices (Dong et al., 2024; Smalling et al., 2023; Hu et al., 2016; Wei et al., 2018; Hu et al., 2021; Podder et al., 2021; Zhang et al., 2024; Brusseau et al., 2020; George and Dixit, 2021; Hu et al., 2023). In many cases, researchers collect their own data to ensure consistency and quality, or they utilize federally curated datasets, such as those from the EPA Unregulated Contaminant Monitoring Rule (UCMR) (Hu et al., 2016; Rosenblum et al., 2024; Guelfo and Adamson, 2018), which have a consistent data reporting format. Consequently, it remains unclear whether conclusions drawn from one dataset are generalizable or comparable to those derived from others.

Numerous studies have underscored the importance of publicly accessible environmental datasets for addressing global challenges such as climate change, pollution, biodiversity loss, and resource management (Liddie et al., 2025; Reichman et al., 2011; Hampton et al., 2015; National Academies of Sciences, Engineering, and Medicine, Policy and Global Affairs, Board on Research Data and Information, Committee on Toward an Open Science Enterprise 2018). Environmental contaminant data present unique challenges due to their heterogeneous formats, temporal and spatial data availability, and data quality documentation (Michener, 2015; Wilkinson et al., 2016; Stall et al., 2018). The FAIR principles (Wilkinson et al., 2016), Findability, Accessibility, Interoperability, and Reusability, have been widely adopted to promote standardized practices for data sharing across research domains (National Academies of Sciences, Engineering, and Medicine, Policy and Global Affairs, Board on Research Data and Information, Committee on Toward an Open Science Enterprise 2018; Mons et al., 2017). Applying FAIR principles to environmental contaminant data can enable innovation because of increased data inventories, systemic inequity identification, and overall strengthen stakeholder participation (Liddie et al., 2025; Quay et al., 2022).

To assess adherence to FAIR principles, FAIR and domain-specific checklists have been developed (Wilkinson et al., 2019), some using rule-based scoring systems or ontology-driven assessments and automated metadata extractors (Sansone et al., 2019; Da Silveira et al., 2024). These tools have demonstrated success in evaluating data in fields such as biomedical research, genomics, and earth sciences (Lamarre et al., 2025; Wilkinson et al., 2018; Qiu et al., 2024; Lia and Colella, 2023; Nault et al., 2023; Van Buggenhout et al., 2025). Recent advancements in Large Language Models (LLMs), such as GPT, Claude, and Gemini, have opened new possibilities for addressing many of these challenges. LLMs, trained on extensive textual data, possess the ability to extract, summarize, and classify information across unstructured documents (Persson, 2024; Brown et al., 2020). Their generalization capabilities make them well-suited for synthesizing metadata, identifying inconsistencies, and interpreting complex environmental datasets (Bommasani et al., 2021), which can facilitate FAIR evaluation. Because PFAS datasets often present key metadata inconsistently or only in narrative form, manual assessment can be slow and variable. LLMs help standardize this process by extracting relevant metadata, mapping it to FAIR criteria, and providing transparent, reproducible scoring. Prompt engineering, including techniques like zero-shot, few-shot, and chain-of-thought prompting, enables users to guide LLMs toward highly specific evaluation tasks (Wei et al., 2022; Zhu et al., 2023). Additionally, in-context learning allows LLMs to adapt to domain-specific instructions without requiring retraining, making them scalable tools for supporting automated FAIR assessments and environmental data analysis (Open et al., 2023). These advancements highlight the need to integrate advanced artificial intelligence (AI) into research to reduce manual effort and improve efficiency.

To address these PFAS data gaps, we developed the FAIR+Environmental framework, which extends the FAIR principles by adding two contaminant-specific dimensions: Data Integrability (spatial–temporal completeness and harmonization) and Data Quality (analytical methods and QA/QC indicators). Using this framework, we analyzed over 100 PFAS databases across environmental matrices (groundwater, surface water, drinking water, soil) to assess the current landscape of publicly available PFAS monitoring data in the US. We analyzed PFOA concentrations across states to assess contamination levels relative to its maximum contaminant level (MCL) and identify spatial and temporal patterns. This analysis helps contextualize the current progress in PFAS regulation, policy implementation, and remediation efforts, offering insights into the utility of existing data for future environmental decision-making.

## 2. Methods

### 2.1. PFAS data collection

To assess the availability, quality, and integrability of PFAS contamination data across environmental matrices in the United States, we performed a comprehensive web-based search to compile publicly accessible and agency-reported datasets. We focused on four environmental matrices: groundwater, surface water, drinking water, and soil. Data sources included national databases, multi-state initiatives, and state-specific monitoring programs. All datasets were manually reviewed to retain metadata, documentation, and source links for downstream data assessment. Detailed dataset metadata and source references are provided in Supporting Information (SI), Table S1.

Groundwater PFAS datasets were obtained from 25 individual state agencies as well as nation-wide monitoring programs such as EPA’s UCMR3 and UCMR5, the PFAS Analytical Tools, the Environmental Working Group (EWG), and the PFAS Project Lab (PPL) databases. Surface water data were compiled from 47 states datasets as well as federal programs (UCMR3, UCMR5), the PPL database, USGS, San Francisco Estuary Institute (SFEI). Drinking water PFAS data were sourced from national databases (Department of Defense (DoD), the Environmental Working Group (EWG), EWG, PPL) and supplemented with state-level datasets from public health agencies, utility reports, and university-led sampling programs. Soil PFAS data were gathered from a smaller but diverse set of sources, including national compilations such as the PPL and EWG databases, alongside datasets from state agencies, academic institutions, and environmental health departments. Water datasets encompassed all states and the District of Columbia but soil datasets span more about ten states. The compiled data vary in reporting formats, temporal resolution (e.g., year-only to full date), and spatial specificity (e.g., GPS coordinates to facility-level labels).

### 2.2. FAIR+Environmental data evaluation framework

To enable users to efficiently evaluate the sharing degree and data quality of various open environmental datasets, we developed the Fair+Environmental framework, which addresses data sharing and quality. We included six evaluation metrics. Four of these categories are adapted directly from the original FAIR Principles (Wilkinson et al., 2016): Findable, ensures data can be easily located by users and systems; Accessible, evaluates the availability of data under well-defined conditions; Interoperable, assesses whether data can be integrated with other datasets and systems; Reusable, determines whether data is well-documented and suitable for future reuse. Within the Environmental component of the framework, we introduced two new categories (Data Integrability and Data Quality) to address specific challenges associated with contaminant datasets. Data Integrability assesses the potential for merging datasets. It includes two key sub-metrics: Temporal Integrity, which evaluates the sampling date consistency and completeness and Spatial Integrity, which assesses the geographical accuracy and resolution of the sampling site. Data Quality evaluates the reliability and usability of environmental data through four key sub-metrics. Sample Analytical Method examines the documentation and standardization of analytical methods to ensure replicability and consistency. Sample Detection Limit assesses the suitable for the intended analysis (ex. Data imputation for statistical tests unable to handle blanks). Field Quality Control focuses metadata availability during sample collection, such as the use of field blanks and duplicates. Finally, Analytical Quality Control evaluates the quality assurance practices applied during data analysis, including calibration procedures and internal standards (extraction and isotope dilution), to validate the accuracy and precision of results. Together, these metrics provide a comprehensive assessment of data reliability and fitness for use. Detailed descriptions of all metrics and their evaluation criteria are provided in Table 1.

### 2.3. LLM-based FAIR data evaluation

#### 2.3.1. Development of a FAIR assessment tool using LLMs

To evaluate the adherence of environmental datasets to the FAIR (Findable, Accessible, Interoperable, Reusable) principles, we developed a semi-automated assessment framework leveraging Azure OpenAI models. The framework integrates rule-based scoring logic with prompt engineering techniques to simulate expert-level evaluation. Different prompt engineering strategies (e.g., zero-shot, few-shot, and chain-of-thought prompting) were tested to identify the most effective approach for consistent scoring. LLMs are well suited for FAIR assessment because many FAIR sub-criteria require interpreting textual metadata rather than numerical values. Tasks such as determining the presence of persistent identifiers, licensing information, or standardized access protocols align closely with LLM strengths in extraction, classification, and rule-based reasoning. By translating FAIR rubric items into structured prompts, the model replicates the interpretive steps of a human reviewer while providing greater consistency and reducing manual effort.

In this research, LLM involvement was limited to the FAIR assessment; the environmental dimensions (Data Integrability and Data Quality) were evaluated manually using a structured rubric because these criteria require detailed review of heterogeneous file types and case-specific sampling and analytical contexts that are not yet reliably handled by LLMs. Automated LLM-based evaluation of these environmental metrics represents a promising direction for future work.

A scoring rubric was adapted from established FAIR data assessment frameworks, assigning maximum scores to each principle: Findable (17 points), Accessible (10 points), Interoperable (8 points), and Reusable (7 points), for a total of 42 points. Each principle was further broken down into sub-criteria, including identifier type, metadata quality, data access mechanisms, and licensing clarity, following the Australian Research Data Commons (ARDC) (Australian-NZ Data Quality Interest Group Meeting 2023; Australian Research Data Commons-ARDC 2023).

Dataset names and website links were compiled, and each entry was programmatically evaluated through a Python script interfacing with the Azure OpenAI models. We scraped each dataset webpage (title, description, license cues, file formats, download links, text snippet) using Python and passed this extracted content directly to the LLM. The model produced markdown-formatted outputs, which were converted into numerical FAIR scores using a deterministic rule-based parser. This parser applies regular-expression patterns to identify valid markdown table rows for the F, A, I, and R components, and includes fallback handling for missing or malformed rows. Chain-of-thought commentary or other non-table text is automatically ignored, with any parsing anomalies logged and assigned default placeholder values (0 or NA). Outputs exceeding maximum thresholds (e.g., *F >* 17) were automatically flagged and excluded, and a 3-second delay between API calls was implemented to comply with rate limits. Each benchmarking round also underwent a brief human validation step to confirm that flagged entries were true formatting failures rather than false positives, and any outputs failing these criteria were re-evaluated using the same pipeline. More detailed information is provided in SI, Text S1.

To assess the effectiveness and practicality of the proposed LLM-based framework, we benchmarked it against four alternatives: (i) the ARDC FAIR Data Self-Assessment Tool (manual scoring, questionnaire-based), (ii) the F-UJI Automated FAIR Assessment Tool (Lamarre et al., 2025), (iii) a rule-based LLM (zero-shot CoT), and (iv) one-shot, and (v) few-shot CoT LLMs with embedded worked examples to enhance reasoning transparency. For the one-shot CoT approach, we additionally examined how different domain examples influence scoring outcomes. A detailed overview of these methods is provided in Table 2, and the corresponding code for all LLM methods is available in our GitHub repository: https://github.com/JialinDong/LLM-FAIR-PromptEngineering/tree/main.

Lower temperatures yield more deterministic and repetitive outputs, while higher temperatures increase randomness and output diversity (Wang et al., 2020). Prior studies indicate that LLM problem-solving performance is largely stable across temperatures from 0.0 to 1.0 (Renze and Guven, 2024). Accordingly, we selected temperature = 0.2. For each prompting strategy, we conducted multiple evaluation runs and reported the standard deviation to account for the small amount of stochastic variability introduced by the model.

To assess accuracy and robustness, we computed the Root Mean Square Error (RMSE) between predicted scores and ARDC baseline scores, along with standard deviation error bars across datasets. RMSE was calculated separately for each FAIR dimension (F, A, I, R) and for the total FAIR score. Additional error metrics (mean absolute error (MAE), Pearson, Spearman) were also computed to capture score magnitude deviations and rank-order correlations, but RMSE was used to compare assessment methods.

In addition, we assessed reproducibility by executing the same evaluation pipeline using several Azure OpenAI model variants (API version 2024–02–01), specifically: gpt-4o, gpt-4-turbo, gpt-4o-mini, gpt-4, and gpt-3.5-turbo. Each model received identical prompts and datasets, and their FAIR scores were computed to determine the consistency of outputs under a unified scoring rubric. To quantitatively assess reproducibility, we calculated the mean, standard deviation (STD), median absolute deviation (MAD), and coefficient of variation (CV) of the Total FAIR Scores across all model variants. Additionally, pairwise Pearson correlation coefficients were computed to evaluate the alignment of scoring patterns between models. This cross-model analysis enabled us to examine how consistently different LLM variants interpret FAIR principles, and whether architectural differences or optimization strategies (e.g., Turbo vs. Standard) influence the stability or interpretability of FAIR scoring outcomes.

#### 2.3.2. LLM-based FAIR evaluation of PFAS datasets

Using the most effective FAIR assessment method (based on RMSE), we evaluated the FAIRness of PFAS databases within each environmental matrix and in between matrices, and compared multi-state vs. single state data sources. Six multi-state data sources were selected: DoD, EWG, PPL, UCMR3, UCMR5, and USGS. These datasets span a broad geographic range across the United States and include multiple matrices: groundwater (GW), surface water (SW), drinking water (DW), and soil. Some datasets included more than one matrix (e.g., PPL includes DW, GW, SW, and soil). To build a control group for comparative analysis, we selected ten single-state data sources representing individual state environmental monitoring programs. These include Alaska DEC, GAMA (California), NMED groundwater (New Mexico), NMED drinking water (New Mexico), ADEQ (Arizona), CDPHE (Colorado), Maine DWP, DSHS (Texas), Virginia Department of Health, and RWRD (Arizona). The selected datasets were stratified to reflect a similar distribution of source types as observed in the multi-state group, ensuring the inclusion of DW, GW, SW, and soil sources where available. This stratified selection ensured that comparisons were not biased by disproportionate representation of a particular source category.

### 2.4. Data integrability and data quality evaluation

#### 2.4.1. Evaluation criteria

Data integrability and data quality were evaluated using both a traditional questionnaire-based approach and an LLM-based automated evaluation. The evaluation rubric (Table 3) defines sub-metric and criteria with associated point values. Each sub-metric is assigned a score based on the presence, completeness, or precision of the corresponding dataset attributes. The maximum possible score for each category is 6, allowing for semi-quantitative comparison across datasets. These Environmental metrics were evaluated independently, followed by a structured manual scoring rubric to maintain consistency with contaminant-specific assessment needs.

A questionnaire-based evaluation was conducted manually for each PFAS database by human reviewers. Reviewers directly examined the dataset files and associated documentation to assess metadata completeness.

### 2.5. PFAS data processing and analysis

#### 2.5.1. Data processing

Following data acquisition, each dataset underwent a systematic processing pipeline to ensure harmonization across varied formats and sources. The raw data were obtained in heterogeneous formats, including Excel, comma-separated values (CSV), tabular data embedded within online dashboards, ArcGIS shapefiles, web-based interactive maps, and PDF technical reports. For geospatial and map-based datasets, data extraction was conducted using a combination of browser-based scraping tools and geospatial data conversion techniques to access and standardize the underlying tabular information. In cases where data were presented in PDF format, tabular information was manually extracted and digitized using optical character recognition (OCR) or PDF-to-spreadsheet tools, with manual verification applied as needed to ensure accuracy.

For datasets already available in tabular format, a standardized data cleaning protocol was implemented. This included filtering out non-relevant records (e.g., metadata-only entries or administrative logs), removing duplicated entries, correcting unit inconsistencies, and dropping non-informative or redundant columns. A uniform data schema was established to facilitate harmonization across sources, specifying required attributes such as sampling location, temporal information (e. g., sampling date), PFAS analyte identifiers, concentration values, units, and source metadata. Each dataset was reformatted in accordance with this schema to ensure interoperability.

For non-tabular sources such as GIS layers, interactive mapping platforms, and PDF documents, equivalent structured representations were derived and conformed to the standardized schema. Geospatial coordinates were extracted when available, while location descriptions (e.g., facility name, county, or state) were normalized using a consistent set of naming conventions and geographic references.

#### 2.5.2. Data analysis

Data analysis was performed in Python. All collected PFAS data sources were consolidated into a unified, comprehensive dataset. We analyzed the quantity and geographic distribution of PFAS data across four environmental matrices, groundwater, surface water, drinking water, and soil, for each U.S. state. To visualize spatial patterns, U.S. state boundary shapefiles were obtained from the U.S. Census Bureau and used to generate choropleth maps that illustrate the number of PFAS datasets available per state. This enabled the assessment of both data availability and regional coverage in varying environmental contexts. Data processing, mapping, and statistical analysis were performed using Python libraries including ‘pandas’ for data manipulation, ‘geopandas’ for geographic data handling, ‘matplotlib’ for visualization, and standard statistical packages for basic descriptive analysis.

## 3. Results and discussion

### 3.1. Number of PFAS datasets in the U.S

**Fig. 1.**
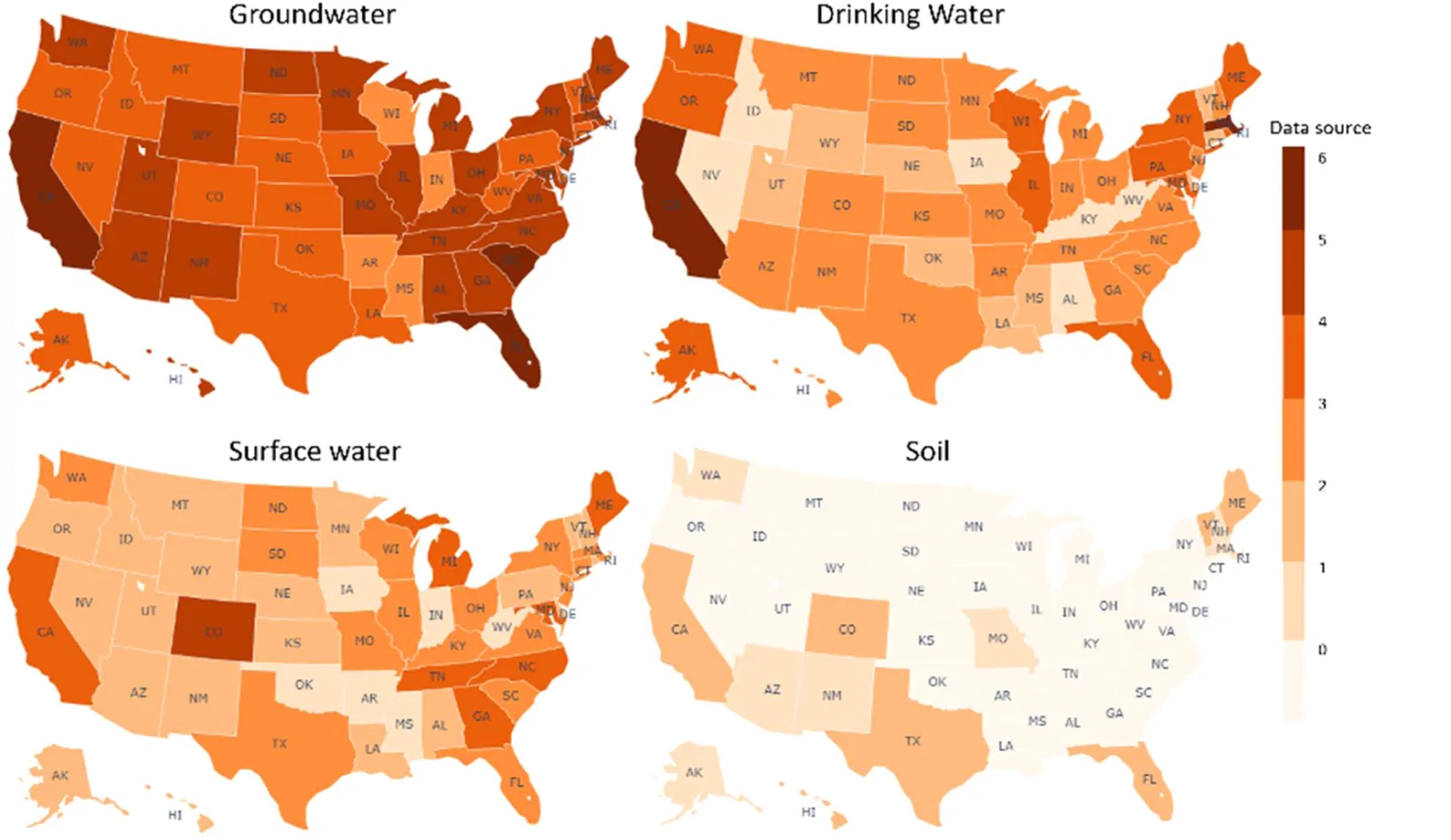
The number of current public data sources for PFAS in groundwater, drinking water, surface water and soil in U.S.

The availability of publicly accessible PFAS monitoring data varies considerably across environmental matrices in the United States (Fig. 1). Groundwater exhibits the most comprehensive coverage, with data reported in all 50 states primarily via nation-wide programs and a substantial number of state-level sources. Drinking water also shows widespread data availability, although regional gaps remain, particularly in the central and western U.S. In contrast, surface water data are more limited, with significant geographic disparities and fewer states have widespread geographic coverage. Soil represents the least documented matrix, with only a small subset of states reporting PFAS data and generally limited temporal and analyte scope.

The distribution of groundwater data sources is relatively comprehensive across the United States, with high coverage in the Northeast (e.g., Pennsylvania, New Jersey), Midwest (e.g., Illinois, Ohio), and parts of the West (e.g., California, Washington). Four major public datasets, UCMR3, UCMR5, PFAS Analytic Tools, and EWG, cover all states, ensuring each state has at least one publicly available database. Additionally, 32 states and Puerto Rico offer state-level or local datasets, with California, Virginia, West Virginia, New Mexico, Michigan, Florida, North Carolina, South Carolina, and New Jersey having the most extensive coverage.

PFAS data availability for drinking water is most concentrated in the Northeast (e.g., Massachusetts, New York), the upper Midwest (e.g., Michigan, Wisconsin), and coastal states such as California and Oregon. This pattern indicates more comprehensive monitoring efforts, likely due to higher population density or known contamination sources. In contrast, coverage remains sparse across much of the Central United States, reflecting regional disparities that may hinder comprehensive assessments of human exposure risks. Nationally, drinking water datasets are available for 44 of the 50 states and Puerto Rico, with six states lacking PFAS drinking water sampling data. Major national programs, including those led by the DoD and EWG, provide data for 40 states.

PFAS monitoring in U.S. surface water exhibits moderate variability across states, with higher data density observed in Colorado, California, and several northeastern states. These patterns reflect targeted efforts to assess PFAS contamination in watersheds likely impacted by PFAS-known and likely contaminated sites near industrial or military activities. In contrast, large portions of the Midwest, the South, and the central Plains show limited or no data coverage, highlighting significant regional gaps in surface water monitoring. Nationally, datasets reporting PFAS levels in surface water are available for 31 of the 50 states, with 20 states lacking any publicly available PFAS data. The primary data sources are the UCMR3 and UCMR5 programs from the USEPA, which cover 23 states combined.

Among the four environmental matrices analyzed, soil data is especially lacking. Only 14 states, including Vermont, Massachusetts, Hawaii, Maine, Florida, Colorado, Arizona, New Mexico, California, and Texas, have reported PFAS data in soil, with most states providing only a single data source. A few, such as Florida and several Northeastern states, report more than one.

### 3.2. Performance of FAIR evaluation model

#### 3.2.1. Model performance comparison

Few-shot CoT LLM provided the most accurate and reproducible FAIR scores, making it the most suitable model for approximating expert-based FAIR evaluations. It achieved the lowest overall prediction error, with a MAE of 13.62 and RMSE of 15.97 for the total FAIR score (Table 4). It also outperformed other models across most individual FAIR dimensions. In the Interoperability category, the model achieved moderate correlation with ARDC scores (Pearson = 0.43; Spearman = 0.33), indicating its ability to preserve both score magnitude and relative rank. This improvement stems from embedding few-shot examples and chain-of-thought reasoning, which enhanced clarity, consistency, and interpretability. Providing examples based on the EPA UCMR3 PFAS dataset and Natural Earth (NE) data enabled step-by-step reasoning, reducing hallucinations and improving reliability. Compared with rule-based LLMs and rigid tools such as F-UJI, the few-shot CoT approach more effectively handled incomplete or ambiguous metadata, showing greater flexibility and robustness across heterogeneous datasets. A comparison of one-shot CoT LLMs highlights the importance of domain relevance and diversity of examples. The EPA-based model aligned more closely with expert scores (RMSE = 19.51 vs. 23.48), underscoring the value of domain-specific examples, while the NE-based model, though less precise, benefited from more examples, improving robustness. These findings suggest that effective prompt engineering requires both domain-relevant and diverse examples.

#### 3.2.2. Reproducibility of LLM-Based FAIR assessment

To assess the consistency of FAIR evaluations across different Azure OpenAI model variants, we computed the Total FAIR Scores using the same dataset and prompt (few-shot CoT LLM) across five versions: gpt-4o, gpt-4-turbo, gpt-4o-mini, gpt-4, and gpt-3.5-turbo (Table 5). The results demonstrated strong alignment in scoring trends across all models (average scores ranging 52–55.3). Among the models, gpt-4o exhibited the lowest variability, with a standard deviation (STD = 14.6) and the lowest coefficient of variation (0.26), indicating high scoring stability. Conversely, gpt-4-turbo showed slightly higher dispersion (STD = 19.3; CV = 0.37), reflecting its broader output range. Correlation analysis further supported high reproducibility, with Pearson coefficients exceeding 0.80 between all model pairs and reaching as high as 0.96 between gpt-4 and gpt-4-turbo. These findings suggest that FAIR assessments are robust to architectural and optimization differences among LLMs, with minimal deviation in scoring consistency, thereby reinforcing the reliability of LLM-based FAIR evaluations for environmental datasets.

In addition, model robustness in adhering to the required markdown output format was consistently high across the evaluated Azure OpenAI variants. gpt-4o and gpt-4o-mini produced the fewest formatting errors (*<*2 %), while older models such as gpt-4 and gpt-3.5-turbo exhibited slightly higher rates of malformed tables (SI, Table S2).

Although the few-shot CoT LLM improves scoring consistency and reproducibility, its performance remains limited by the completeness and clarity of dataset metadata. When essential fields, such as identifiers, licensing information, or data source notes, are missing or ambiguously reported, the model may produce uncertain or overly conservative evaluations that require manual verification. Therefore, results generated by the LLM should always be validated by domain experts, as LLM-based FAIR assessments serve as decision-support tools rather than replacements for expert review.

### 3.3. Evaluating FAIRness of PFAS databases using LLM-based model

#### 3.3.1. Best model (few-shot CoT LLM gpt-4o) performance on U.S. PFAS database

**Fig. 2.**
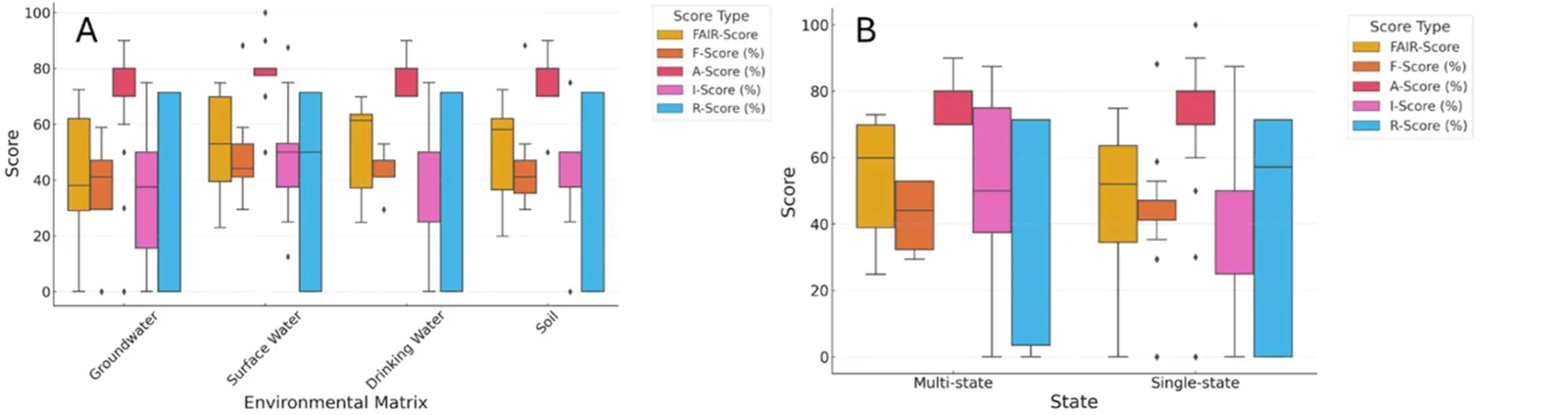
Distribution of FAIR principles scores by Environmental Matrix (Panel A) and state-level data (Panel B).

Using the best-performing model, the few-shot CoT LLM (gpt-4o), we streamlined FAIR evaluations for the U.S. PFAS database and analyzed results by data class and geographic scope. Among the four major data classes, Surface Water datasets achieved the highest average FAIR-Score (53.57 %), driven by strong performance in Accessibility (78.40 %) and Interoperability (48.50 %) (Fig. 2). Drinking Water datasets also performed well in Accessibility (77.7 %) but showed lower scores in Interoperability and Reusability, resulting in a moderate FAIR-Score (52.6 %). In contrast, Groundwater datasets exhibited the lowest FAIR-Score (45.2 %), primarily due to consistently lower scores in Findability and Reusability. Soil datasets fell in between, with balanced scores across all dimensions.

Multi-state datasets demonstrated notably higher FAIR-Scores (multi-state 55.5 % vs. single-state 48.93 %), with improvements observed across all dimensions, especially Interoperability (multi-state 54.5 % vs. single-state 41.6 %) and Reusability (multi-state 47 % vs. single-state 37.1 %). These findings suggest that datasets covering multiple states tend to be more interoperable and reusable, potentially due to more standardized formats, collaborative reporting protocols, or broader data integration efforts. Detailed statistical analyses and the complete FAIR evaluation scores for all PFAS datasets are provided in SI, Tables S3-S6.

An evaluation of single-state datasets revealed substantial variability in FAIR-Scores (SI, Figure S2), with medians ranging from 17.1 to 73.1. Michigan (73.08), Iowa (72.3), and South Dakota (69.8) achieved the highest FAIR-Scores, supported by strong Accessibility, Interoperability, and relatively good Reusability. In contrast, Kentucky (17.1), Idaho (24), and Missouri (25.7) exhibited the lowest scores, largely due to poor Findability and consistently low Reusability, often at 0 %, despite moderate Accessibility. Accessibility and Interoperability were generally higher across most states, while Reusability remained a consistent weakness, highlighting a gap in dataset documentation and licensing for reuse. The number of available datasets for each state varied, with Colorado having the most (7), followed by California (6) and Florida (5). States with more datasets showed more stable distributions, while states such as Idaho and Kentucky (with only one or two datasets) displayed greater variability and lower overall scores.

Among scientific articles with environmental monitoring, research citations are concentrated in states like California and New York, with limited activity in central and Mountain West regions, but show no strong correlation with FAIR scores or data quality, highlighting a gap between data availability and research focus. Detailed information is provided in SI, Text S2 and Figure S1.

PFAS datasets show substantially lower FAIR compliance (SI, Figures S3-S4) compared to non-PFAS databases, such as the New Zealand Geomagnetic Database (91 %) and the New Zealand National Groundwater Monitoring Programme (88 %) (Australian-NZ Data Quality Interest Group Meeting 2023; Nationally Significant Collections and Databases 2025). PFAS datasets need significant FAIRness improvement to enable broader reuse in environmental research and policymaking. Coordinated national or regional data management strategies are essential to promote harmonization and interoperability, particularly for datasets aggregated across multiple jurisdictions or sources.

To address the gaps identified in the FAIR assessment, we recommend that environmental data repositories, including those of state and federal agencies, laboratories, and academic researchers, adopt the FAIR+Environmental checklist as part of routine data reporting. This includes standardized documentation of detection limits, analytical methods (e.g., EPA 1633/537.1), units, geospatial metadata, and QA/QC information, all of which are essential for data integrability and scientific reliability but are often missing in current PFAS datasets. Because the scoring relies only on publicly reported metadata, missing public documentation reflects reporting gaps rather than actual absence of data. To support practical implementation, the LLM-based FAIR+Environmental pipeline can be used as an automated QA/QC tool during data publication or submission to a long-term repository or dashboard. In practice, the LLM can rapidly flag missing metadata fields, inconsistent units, absent method documentation, or incomplete licensing information, allowing data providers to correct issues before dataset release. This workflow offers a feasible path for agencies and researchers to enhance dataset FAIRness, harmonize reporting practices, and improve the long-term value of PFAS monitoring data for research and policy. LLM-based FAIR assessments should be treated as support tools, with results verified by environmental experts.

#### 3.3.2. Data integrability and data quality evaluation

Based on data integrability, drinking water datasets showed the highest consistency, with scores tightly clustering around 83 %. In contrast, surface water and soil datasets exhibit a much wider distribution, 20–100 % and 30–100 %, respectively. Groundwater datasets scored 50–100 %. These patterns suggest that while drinking water datasets might be more easily merged, groundwater, surface water, and soil datasets require greater harmonization to enable reliable comparability.

Based on data quality, drinking water and groundwater datasets show consistently lower median scores, ranging 33–50 %. The small range indicates datasets follow similar standards but have limited reporting quality, suggesting that such datasets may not always be sufficiently reliable for research and detailed environmental assessments. In contrast, surface water and soil datasets achieved higher median data quality scores (~67 %), but with greater variability (0–100 %). This broad distribution suggests that while some surface water and soil datasets are of excellent quality, others lack data quality information. We also compared multi-state vs. single-state datasets and found notable differences in integrability. Single-state datasets had higher integrability (mean 70 %) compared to multi-state ones (mean 58.3 %), with a wider spread among single-state entries. Both categories showed similarly low data quality, with mean values near 28 %, suggesting that geographic scope does not strongly influence the documentation or transparency of data quality.

**Fig. 3.**
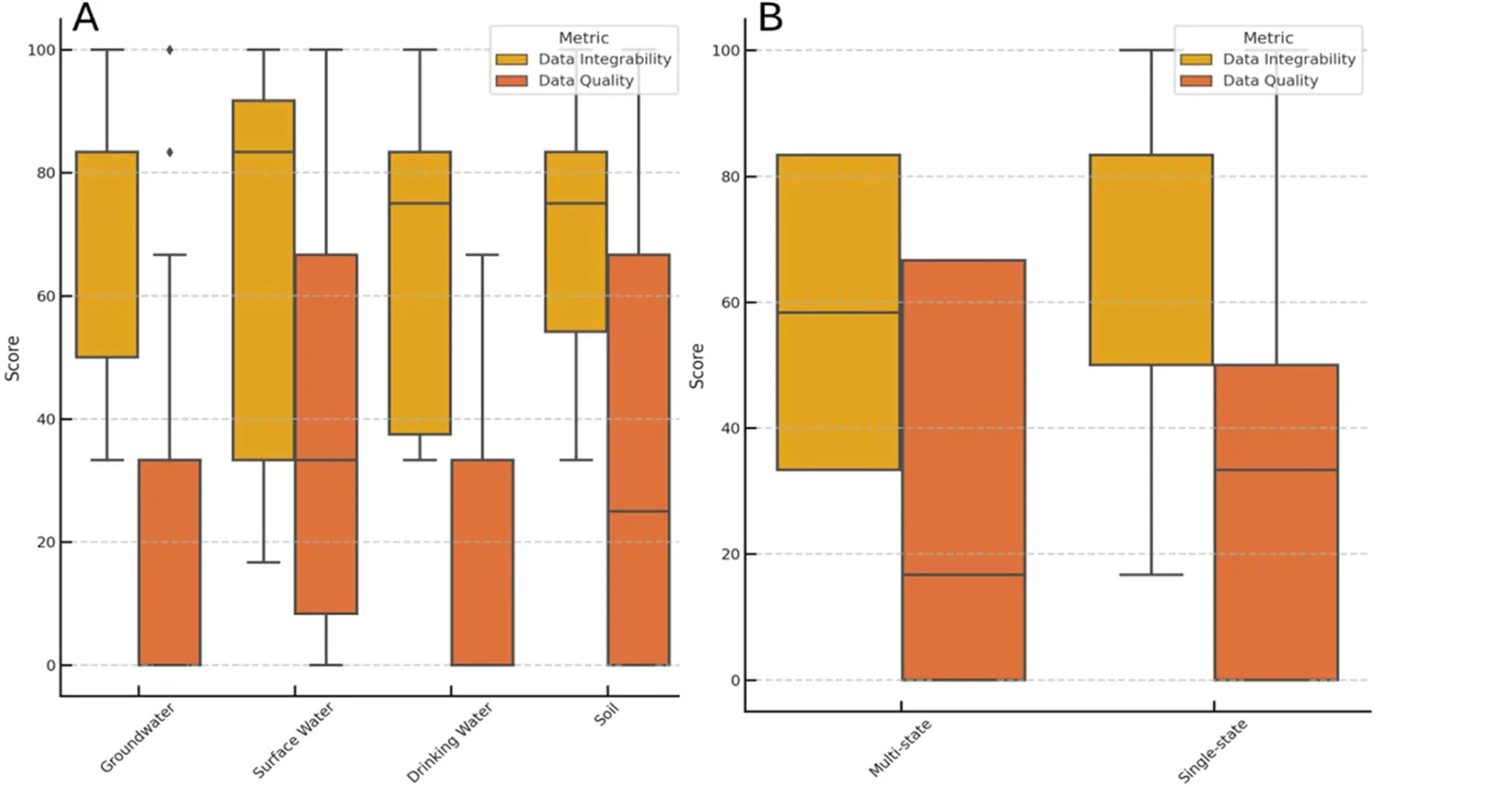
Assessment of data integrability and data quality for PFAS datasets in different environmental matrices: groundwater (39 datasets), drinking water (26 datasets), surface water (31 datasets), and soil (14 datasets).

Moreover, correlation analysis showed that data integrability (*r* = 0.17) and data quality (*r* = 0.10) is only weakly associated with FAIR-Scores, revealing a core limitation of standard FAIR principles when applied to environmental monitoring data. A dataset may score well on FAIR yet still lack essential elements (analytical methods, detection limits, spatial precision, or QA/QC details) critical to environmental contaminants and other constituents that determine its scientific reliability and usefulness for exposure or regulatory assessments. This mismatch appears across matrices: drinking water data are easier to merge but often lack methodological transparency, whereas surface water and soil datasets contain richer detail but remain inconsistent and difficult to integrate. Thus, FAIR alone cannot ensure the robustness of environmental contaminant datasets and should be supplemented with domain-specific quality and integrability criteria. The FAIR+Environmental framework is especially important for datasets used in cross-state comparisons, regulatory decisions, and exposure modeling, where methodological transparency and data reliability are critical. (Fig. 3)

### 3.4. U.S. PFAS concentration analysis

#### 3.4.1. PFAS concentration in groundwater

**Fig. 4.**
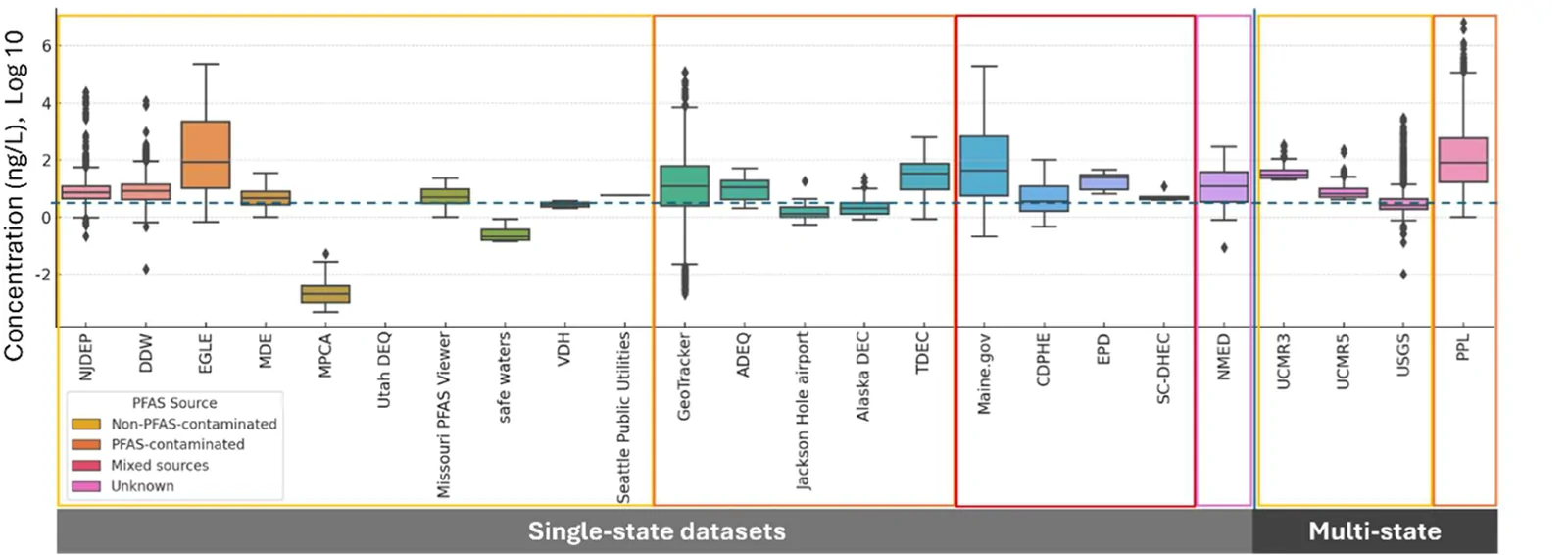
Groundwater PFOA Concentration (ng/L) Distribution by Data Source (log-scaled). The horizontal blue dashed line represents a concentration of 4 ng/L. The vertical blue line separates single-state (left) and multi-state (right) data sources. Sources and sample counts (years): Arizona Department of Environmental Quality (ADEQ) (524, 2013–21), Alaska Department of Environmental Conservation (Alaska DEC) (58, N/A), Colorado Department of Public Health & Environment (CDPHE) (968, 2016–21), California Division of Drinking Water, GAMA/DDW (8360, 2016–23), Michigan Department of Environment, Great Lakes, and Energy (EGLE) (1842, 2017–22), Georgia Environmental Protection Division (EPD) (271, N/A), California Water Boards GeoTracker Program (GeoTracker) (3166, 2016–23), Jackson Hole Airport PFAS Groundwater Sampling Program (185, N/A), Maryland Department of the Environment (MDE) (759, 2021–22), Minnesota Pollution Control Agency (MPCA) (642, N/A), Maine Department of Environmental Protection (Maine.gov) (9643, 2007–23), Missouri Department of Natural Resources PFAS Viewer (449, 2013–23), North Carolina Department of Environmental Quality (NC DEQ) (58, 2019–21), North Dakota Department of Environmental Quality (NDDEQ) (25, N/A), New Jersey Department of Environmental Protection (NJDEP) (29,219, 2019–23), New Mexico Environment Department (NMED) (104, 2020–22), Ohio Environmental Protection Agency (Ohio EPA) (3340, 2019–21), PFAS Project Lab (PPL) (1589, N/A), Ambient Surface Water PFAS Project, South Carolina DHEC (46, 2020), Seattle Public Utilities (3, 2018), Tennessee Department of Environment & Conservation (TDEC) (21, 2018–22), EPA Unregulated Contaminant Monitoring Rule 3 (UCMR3) (23,664, 2013–16), EPA Unregulated Contaminant Monitoring Rule 5 (UCMR5) (20,056, 2023–24), United States Geological Survey (USGS) (2244, 2016–22), Utah Department of Environmental Quality (Utah DEQ) (586, 2013–16), Virginia Department of Health – Office of Drinking Water (VDH) (24, N/A), and JBPHH Safe Waters Program (Safe Waters) (26, 2023–24).

PFOA and PFOS were the most frequently reported analytes across different data sources, followed by PFBS, PFNA, PFHxS, PFHpA, and PFHxA. Given the high detection frequency of PFOA and existence of its MCL of 4 ng/L, we selected this analyte to illustrate the distribution of PFAS concentrations. Statistical analysis of groundwater PFOA concentrations shows notable differences across data sources. Known PFAS-contaminated sources show higher medians, wider ranges, and more outliers than non-PFAS-contaminated sources (Fig. 4). Mixed and unknown contamination sources also display broad distributions. Multistate sources have greater variability and higher upper tails, reflecting diverse sampling regions, while single-state datasets are narrower, capturing localized contamination patterns. Across all sources, PFOA concentrations ranged from 0.2 to 6570,000 ng/L, reflecting both widespread low-level presence and extreme hotspots in source contamination zones. Withing these source contamination zones, the PPL dataset reported the highest maximum (6570,000 ng/L) and mean concentrations (17,785 ng/L). Michigan Department of Environment, Great Lakes, and Energy (EGLE) exhibited the widest concentration range (0.67 to 230,000 ng/L) and the highest median (83.5 ng/L), suggesting consistently elevated levels. Maine.gov contributed the largest dataset with moderate contamination (median 41.7 ng/L). In contrast, Minnesota Pollution Control Agency had the lowest median concentrations (~30.0 ng/L) and narrower ranges. Data distribution is described in SI, Text S3. These results reflect regional variation in PFOA contamination and highlight the need for consistent monitoring approaches.

Groundwater PFOA concentrations differed based on known PFAS contamination vs. no known PFAS contamination. For the data sources without no known PFAS contamination, none had median PFOA concentrations above its MCL of 4 ng/L (Title XIV of the public health service act: safety of public water systems (safe drinking water act) 1994) but 22 % of observations exceeded this regulation. States and territories with mean PFOA concentrations above 4 ng/L included California, Colorado, Massachusetts, Michigan, Northern Mariana Islands, New Jersey, and West Virginia. These findings indicate that groundwater from non-PFAS-contaminated sources is generally safe, with the majority of PFOA concentrations well below regulatory limits. In contrast, 88 % of data sources with known PFAS contamination had median above 4 ng/L, and 58 % of the observations were above the MCL. Nevada showed the most severe contamination (up to 6.57 mg/L). States and territories with median concentrations higher than the national average of medians (~500 ng/L) included Arkansas, Louisiana, Nebraska, and Puerto Rico. Data sources with known PFAS contamination sources not only have higher median levels in most states but also exhibit extreme upper-bound values that are orders of magnitude greater than their non-contaminated counterparts. Descriptive statistics of groundwater PFOA concentrations for all states (SI, Figures S5–6).

Among no known PFAS contamination, none of the states showed statistically significant annual monotonic trends. Both Ordinary Least Squares (OLS) regression and the Mann–Kendall (MK) test consistently indicated no change. Using state/territory-level monthly medians, two states exhibited significant increasing trends, one showed a decreasing trend, and 53 demonstrated no significant change over the study period (SI, Table S6). Connecticut and New Jersey increased (+0.049 and +0.009 ng L^−1^ per month; τ = 0.157 and 0.102), while Colorado decreased (−0.314 ng L^−1^ per month; τ = −0.116).

#### 3.4.2. PFAS concentrations in surface water

**Fig. 5.**
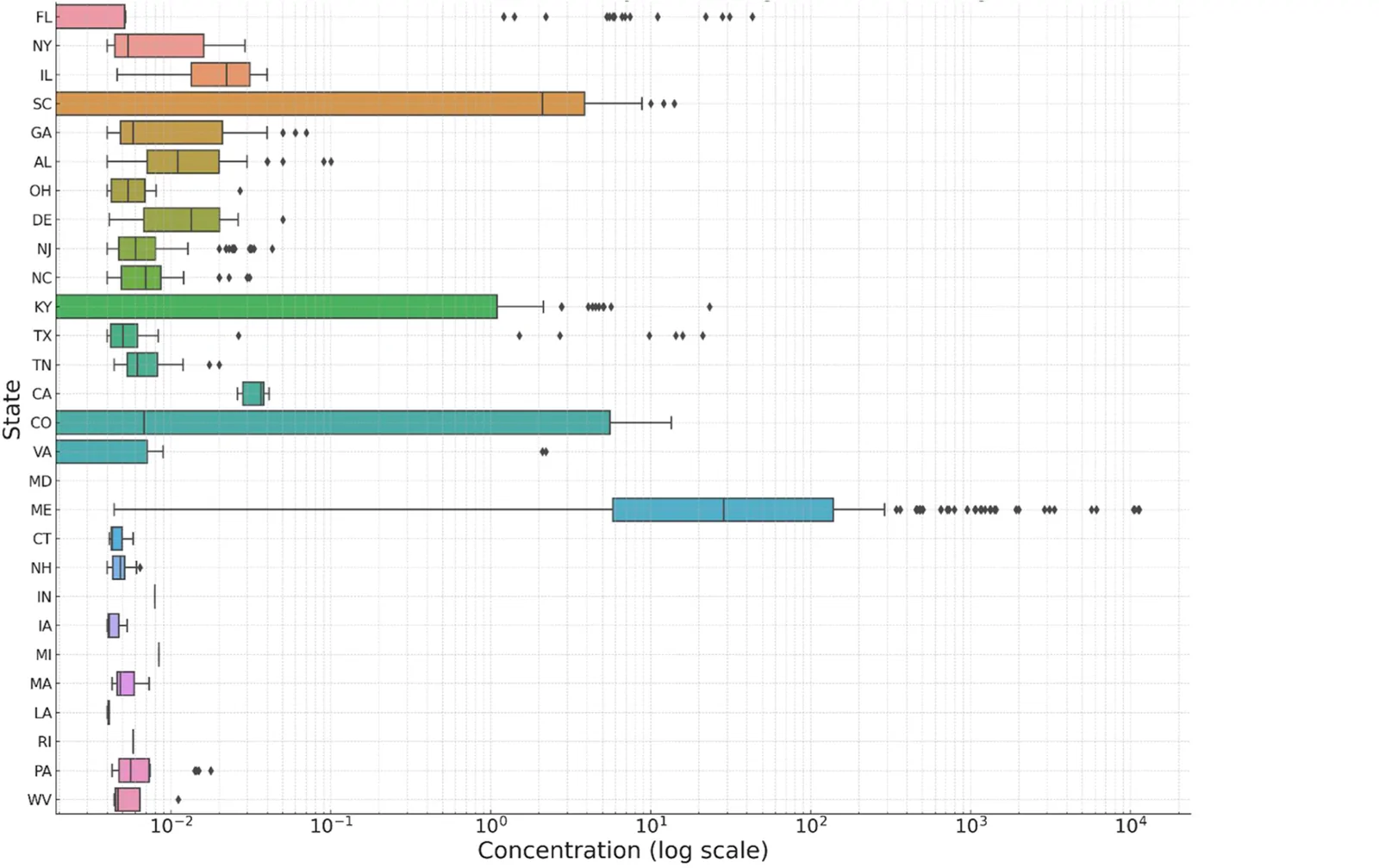
Surface water PFOA concentration (ng/L, Log10 scaled) distribution by state.

PFOA, PFOS, PFHxS, and PFBS were the most commonly reported analytes in surface water, followed by PFHpA, PFNA, PFHxA, PFBA, PFPeA, and GenX. As in groundwater, we selected PFOA to represent the distribution of PFAS concentrations because of his high frequency of detection. Based on all observations, 637 samples (32 %) exceeded 4 ng/L despite low median concentrations. Multi-state drinking water databases such as UCMR3 and UCMR5 reported lower concentration levels with relatively narrow ranges. Sites with known PFAS contamination showed higher medians and wider distribution than no-known PFAS contaminated sites. Data distribution described in SI, Text S4. Maine had the highest median PFOA concentration (28 ng/L) and the highest recorded maximum, likely reflecting mixed known PFAS contamination status. Other states, including South Carolina, Kentucky (sampling from known contaminated sources), and Colorado showed wide ranges of PFOA concentration distributions, suggesting significant variability in contamination levels across sampling sites within these states. Only one of these states had a median concentration above 4 ng/L, underscoring that while extreme contamination events exist, most states report comparatively lower median levels. PFOA data distribution for all data sets (SI, Figure S7-S9) (Fig. 5).

Surface water temporal analysis of state-level annual median PFOA concentrations from sites without known PFAS contamination showed no significant (Ordinary Least Squares (OLS), Mann–Kendall (MK) tests) monotonic trends. Monthly trend analysis of state-level median PFOA concentrations from non–PFAS-contaminated sources showed that 24 (80 %) states exhibited no significant change (SI, Table S7). However, four states demonstrated significant decreasing trends: Alabama (OLS slope = –2.1 × 10^−4^ ng/L per month, τ = –0.50), New Jersey (–2.0 × 10^−4^, –0.44), New York (–1.9 × 10^−4^, –0.42), and South Carolina (–5.7 × 10^−2^, −0.52). Two states showed evidence of increasing trends: New Hampshire (1.2 × 10^−4^ ng/L; τ = 0.36) and Virginia (3.2 × 10^−4^, *p* = 0.010; τ = 0.33).

#### 3.4.3. PFAS concentrations in drinking water

**Fig. 6.**
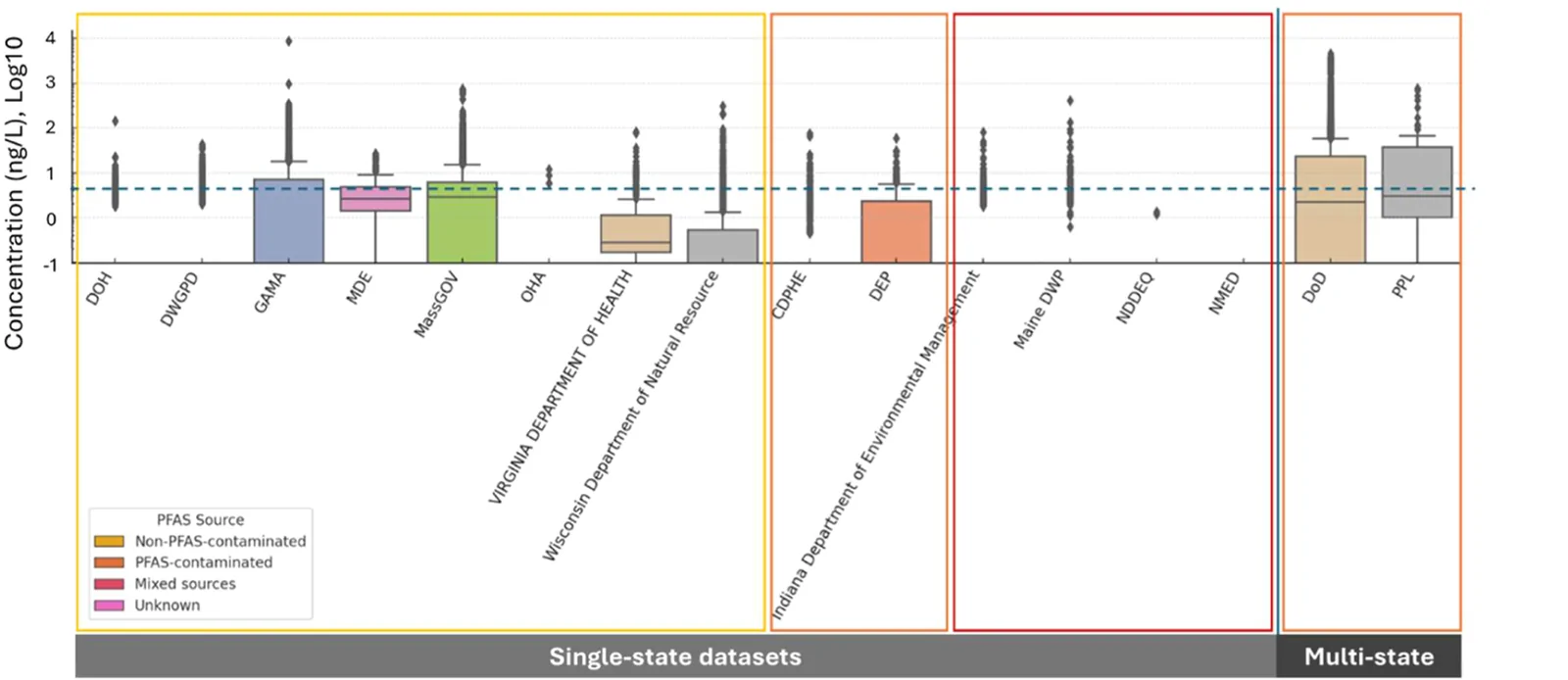
Drinking Water PFOA Concentration Distribution by Data Source (log-scaled). The horizontal blue dashed line represents a concentration of 4 ng/L. Sources and sample counts: Washington State Department of Health (DOH, 1993), Vermont Drinking Water and Groundwater Protection Division (DWGPD, 2068), California Groundwater Ambient Monitoring and Assessment Program / Division of Drinking Water (GAMA/DDW, 23,842), Maryland Department of the Environment (MDE, 228), Massachusetts Executive Office of Energy and Environmental Affairs Public Water Supply Database (MassGOV, 19,906), Oregon Health Authority (OHA, 195), Virginia Department of Health – Office of Drinking Water (296), Wisconsin Department of Natural Resources (3467), Colorado Department of Public Health & Environment (CDPHE, 713), Pennsylvania Department of Environmental Protection (DEP, 412), Indiana Department of Environmental Management (2797), Maine Drinking Water Program (Maine DWP, 721), North Dakota Department of Environmental Quality (NDDEQ, 137), New Mexico Environment Department (NMED, 24), U.S. Department of Defense PFAS Sampling Program (DoD, 14,542), and PFAS Project Lab (PPL, 68).

Statistical analysis of drinking water PFOA concentrations reveals notable variation across data sources (Fig. 6). Multi-state datasets with known PFAS contamination showed higher concentrations and variability than datasets of sites without known PFAS contamination (T-test, *p <* 0.05). Notably, all 16 data sources had median concentrations below the MCL of 4 ng/L. Despite the low medians, 24,166 individual samples (33.8 % of total observations) exceeded the 4 ng/L threshold, indicating that elevated PFOA levels still occur frequently enough to raise public health concerns. Data distribution described in SI, Text S5.

Across all drinking water samples (*n* = 71,449), 33.8 % were above the MCL of 4 ng/L (SI, Figures S10–S12). Nine out of 42 states (21 %) had medians exceeding 4 ng/L, including Arkansas (9.6 ng/L), Delaware (4.15 ng/L), Illinois (4.6 ng/L), Kansas (23.15 ng/L), Pennsylvania (5.1 ng/L), Rhode Island (6.3 ng/L), South Carolina (9.95 ng/L), Tennessee (41.1 ng/L), and Texas (8.8 ng/L). Limiting the analysis to data without known PFAS contamination (*n* = 51,995), 33.1 % exceeded the MCL of 4 ng/L.

Only two out of eight states with data sources without known PFAS contamination had temporal trends on a month-basis. Wisconsin showed an increasing trend (slope = +0.065 ng/L per month, τ = 0.38), while Massachusetts exhibited a decreasing trend (slope = −0.140 ng/L per month, τ = −0.33). When all data sources were combined (42 states), significant increases were again observed in Wisconsin (slope = +0.062, τ = 0.37), while significant decreases were evident in Massachusetts (slope = −0.140, τ = −0.33), Michigan (slope = −2.39, τ = −0.43), and Kansas (slope = −1.54, τ = −0.79). Restricting the analysis to PFAS-contaminated sources (41 states) yielded the largest number of significant decreases: Massachusetts (slope = −0.140, τ = −0.33), Michigan (slope = −2.39, τ = −0.43), Kansas (slope = −1.54, τ = −0.79), and Maine (slope = −1.21, τ = −0.29), alongside the consistent increase in Wisconsin (slope = +0.062, τ = 0.37). Overall, Wisconsin exhibited a robust increasing trend across all categories, whereas Massachusetts consistently showed decreasing concentrations. The stronger prevalence of decreasing trends among contaminated sources suggests possible impacts of regulatory interventions or remediation actions in historically impacted areas (trend analysis for all states in SI, Table S8).

#### 3.4.4. PFAS concentrations in soil

**Fig. 7.**
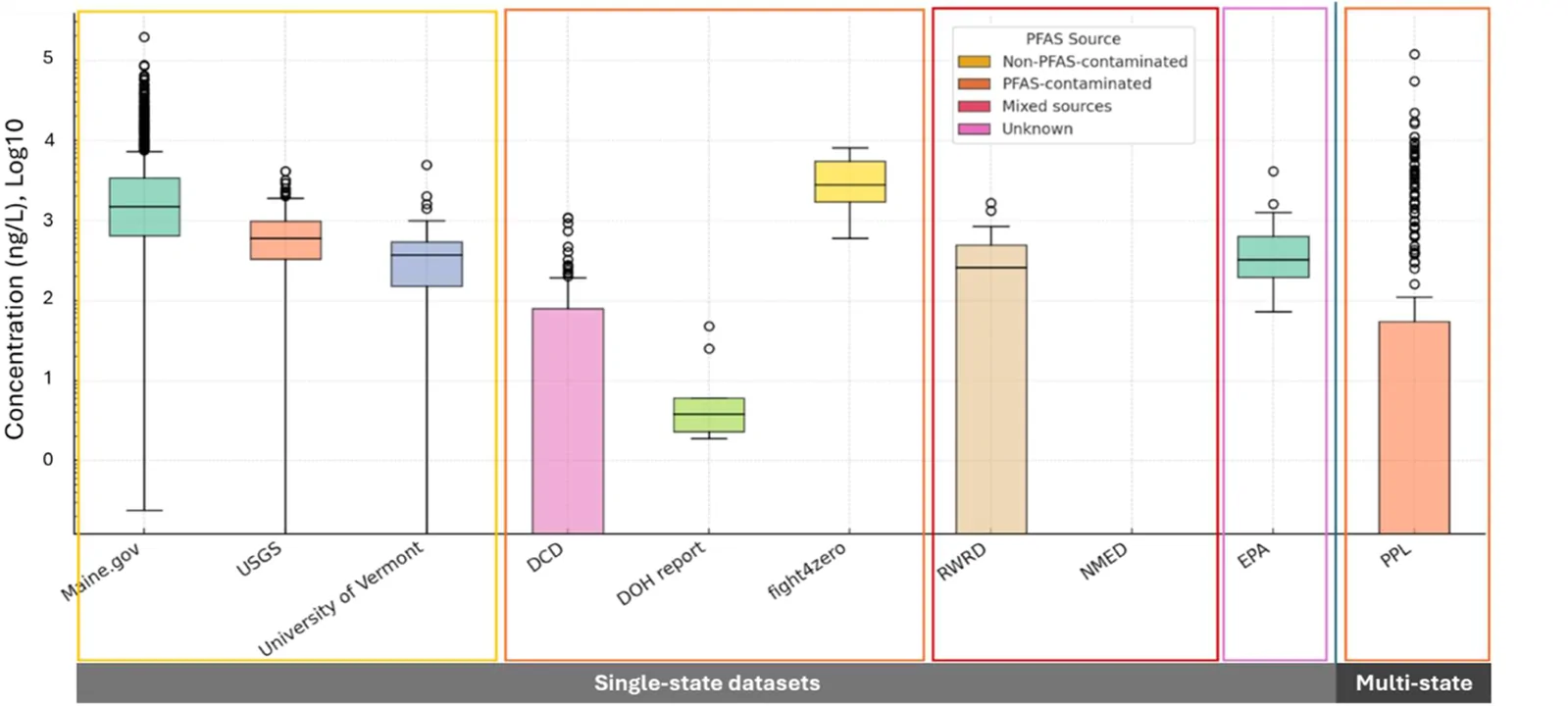
Soil PFOA Concentration Distribution by Data Source (log-scaled). Sources and sample counts (years): Maine Department of Environmental Protection (Maine.gov; 29 PFASs, 421 locations, Nov 2012–Dec 2022), United States Geological Survey – New Hampshire (USGS; 32 PFASs, 100 locations, Feb–Aug 2021), University of Vermont (16 PFASs, 66 locations, June–Aug 2018), Colorado Springs Data Commons / University of Colorado at Colorado Springs (DCD; 9 PFASs, 8 locations, July 2018–June 2019), Fight For Zero – Florida PFAS Community Sampling Program (Fight For Zero; 2 PFASs, 3 locations, Jan–Apr 2019), Pima County Regional Wastewater Reclamation Department (RWRD; 16 PFASs, 1 location, Jan 2020), New Mexico Environment Department (NMED; 23 PFASs, 1 location, Jan 2021), U.S. Environmental Protection Agency – New Jersey Soil PFAS Study (EPA; 12 PFASs, 24 locations, May 2018), and PFAS Project Lab (PPL; 29 PFASs, 21 locations, Aug 2016–Sep 2023).

PFOA was the most frequently detected analyte in soil followed by PFOS and PFHxA. Across all sources, PFOA concentrations ranged from non-detects (*<*1 ng/kg) to maximum values exceeding 10,000 ng/kg (Fig. 7). Larger datasets such as Maine.gov and USGS exhibited broader concentration ranges, with Maine.gov reporting concentrations up to 11,000 ng/kg and USGS up to 9800 ng/kg, and more extensive spatial coverage across over 400 and 100 locations, respectively. In contrast, smaller datasets like DCD (Colorado), Fight For Zero (Florida), and RWRD (Arizona) had more limited geographic representation and narrower concentration distributions. Data distribution is described in SI, Text S6.

We did not observe a clear national trend of increasing or decreasing concentrations over time. States like Maine and California exhibited both high median concentrations and wide ranges, up to ~195,000 and 120,000 ng/kg, respectively, indicating significant local hotspots. In contrast, states such as New Mexico, Hawaii, and Washington showed much narrower ranges, with maximum values of 0, ~45.8, and 96 ng/kg, respectively. However, due to limited time coverage, sampling locations, and uneven data across states, it is difficult to draw definitive conclusions about temporal trends in soil PFAS concentrations. Expanded and standardized soil monitoring is needed to better assess soil temporal trends (distribution across years for each state can be found in SI, Figure S13).

## 4. Conclusions and limitations

We introduced a FAIR+Environmental framework to score PFAS datasets. PFAS datasets exhibit significantly lower FAIR scores than well-established national geoscientific databases, particularly in Reusability, underscoring the need for improved metadata, standardized reporting, and clearer licensing. We suggest FAIR+Environmental framework can be used not only for assessment but also as a practical guide to improve future data collection, validation, harmonization, and public release of PFAS monitoring data. We also developed a semi-automated assessment pipeline using LLMs to evaluate over 100 datasets in groundwater, surface water, drinking water, and soils. Despite their strengths, LLM-based evaluation models face limitations including sensitivity to prompt quality, potential hallucination with incomplete metadata, and higher computational demands, requiring careful prompt design, calibration, and human oversight for reliable use.

Because of the high frequency of detection of PFOA, we used this analyte as indicator of overall PFAS pollution across the continental US and territories. Almost two decades since the adoption of the 2010/2015 PFOA Stewardship Program (USEPA 2016), PFOA concentrations largely have remained consistent, indicating successful reduction of continued PFOA emissions. PFOA levels vary widely across environmental matrices and states, with groundwater and soil acting as major reservoirs of severe contamination, while surface water and drinking water, though generally lower, remain critical exposure routes due to their widespread use. Drinking water median concentrations were below the MCL of 4 ng/L for each state. However, approximately 34 % of individual samples exceeded this threshold, raising significant public health concerns regarding water safety. Because drinking water is one of the primary PFAS exposure media for public health (Sunderland et al., 2019), these findings underscore the need for widespread PFAS treatment across the US. The lack of decreasing PFOA levels over time highlights the widespread need for PFAS removal technologies to protect PFAS exposures across environmental matrices.

## Supplementary Material

MMC2

Supplementary materials

Supplementary material associated with this article can be found, in the online version, at doi:10.1016/j.hazadv.2026.101103.

## Figures and Tables

**Table 1:** FAIR+Environmental framework metrics and evaluation criteria.

Metric	Sub-metric	Detailed description
Data Integrability	Temporal integrity	The degree to which the metadata desk provides date information meets the specification.
	Spatial integrity	The degree to which the metadata provides the spatial information, including whether the spatial range information and other contents are perfect.
Data Quality	Sample analytical method	Documented method used to measure or analyze samples.
	Sample detection limit	The minimum concentration that can be reliably measured (e.g., limit of detection (LOD) or limit of quantitation (LOQ))
	Field quality control	Use of blank samples, duplicate samples, and spike samples during sample collection.
	Analytical quality control	Internal standards, calibration curves, and control charts used during analysis.
Findable	Uniqueness of identifiers	Metadata, data is assigned a globally unique identifier.
	Persistence of identifiers	Metadata, data is assigned a permanent identifier.
	Searchability	To what extent digital resources can be found using web-based search engines.
	Identifier accuracy	The data identifier is included in the metadata, and it matches the identifier provided in the evaluation request.
	Metadata machine retrievability	Metadata is provided in a way that can be retrieved by machines.
Accessible	Access Level Setting	This metric is whether the access level is set to access the requester to a digital object and the access level metadata is machine readable
	Standardized protocols for metadata	This index means that the metadata can be retrieved by the standard communication protocol, given the identifier of the metadata.
	Standardized protocols for data	The scheme for the data URI is based on a shared application protocol and the data is accessible by the provided identifier.
Interoperable	Use of knowledge language	The metadata of an object is provided in a formal knowledge representation language, for example, by at least one of the following mechanisms: resolvable structured data embedded in the login page, resolvable formal metadata (e. g., RDF, JSON-LD) can be accessed through content negotiation, typed links, or sparql endpoints.
	Use of semantic resources	Namespaces for known semantic resources exist in the metadata of objects, e.g., RDF, RDFS, XSD, OWL.
	Data linked to its related entities	This metric is whether the data is linked to its associated entities in order to increase its reuse capability.
Reusable	Provenance of metadata	This metric refers to the metadata associated with the detailed sources. The metric requires the metadata to include information about the source of the data, that is, about the source, history, or workflow of the generated data.
	Existence of licenses	The metadata contains license information represented by the appropriate metadata elements and is machine readable
	Conformity of metadata to community standards	The metric requires metadata to conform to community standards, that is, following community standards related to the target domain and obtain with at least one domain metadata standard
	Conformity of data formats to domain standardization	This metric means that the data is provided in the file format recommended by the target research area. File format refers to the method of encoding the digital information.

**Table 2:** FAIR assessment methods.

Method	Description	Purpose
ARDC FAIR Data Self-Assessment Tool	Manual questionnaire-based tool for self-assessing data against FAIR principles.	Serves as a reference baseline for manual FAIR evaluation practices.
F-UJI Automated FAIR Assessment Tool	Automated tool applying rule-based logic to assess metadata compliance with FAIR principles.	Provides a benchmark for comparison with LLM-based assessment approaches.
Rule-based LLM (Zero-Shot CoT)	LLM prompted with FAIR-related rules, without example inputs.	Establishes a logic-oriented LLM baseline without training examples.
One-shot CoT LLM	Combines one-shot prompting with step-by-step CoT reasoning during evaluation.	Explores how limited examples and explicit reasoning improve performance.
Few-shot CoT LLM	Integrates few-shot examples with CoT reasoning in prompt design.	Aims to optimize both transparency and decision quality in FAIR assessments.

**Table 3:** Scoring rubric for data integrability and data quality evaluation.

Metrics	Sub-metrics and criteria	Score
Data Integrability	1. Temporal integrity	Max weight - 3
	a. Full date (Year, month, day)	3
	b. Year and month, but not date	2
	c. Year only, but not date or month	1
	d. No date information	0
	2. Spatial integrity	Max weight - 3
	a. Latitude, Longitude and no further data processing needed	3
	b. Fuzzy location information (with state, county, city or address, facility name)	2
	c. Fuzzy location information (with state only, no county, city or address, facility name)	1
	d. No spatial information	0
	Total weight	Max weight - 6
Data Quality	1. Sample analytical method	Max weight - 2
	a. Method name included	2
	b. None provided	0
	2. Sample detection limit (DL)	Max weight - 2
	a. Included DL for each target analyte	2
	b. No DL	0
	3. Field quality control	Max weight - 1
	a. Described in metadata or data includes a column mentioning field quality control	1
	b. None provided	0
	4. Analytical quality control	Max weight - 1
	a. Described in metadata or included a column mentioning analytical quality control	1
	b. None provided	0
	Total weight	Max weight - 6

**Table 4:** Root Mean Square Error (RMSE) with Standard Deviation (STD) across FAIR dimensions, comparing model predictions to the ARDC baseline.

Model	Metric	Total FAIR	F	A	I	R
FUJI	RMSE	38.74	34.35	36.2	56.5	39.45
	STD	0	0	0	0	0
Rule-based LLM (Zero-Shot CoT)	Mean RMSE	24.42	27.44	19.6	37.22	34.79
	STD	5.18	5.59	5.56	11.24	6.92
One-shot CoT LLM -EPA	Mean RMSE	19.51	18.5	23.27	24.17	38.34
	STD	12.32	5.84	13.1	20.67	23.42
One-shot CoT LLM -NE	Mean RMSE	23.48	18.76	21.87	37.44	34.98
	STD	6.87	4.59	11.84	14.97	7.52
Few-shot CoT LLM	Mean RMSE	18.13	18.5	22.91	21.43	33.27
	STD	11.83	5.88	9.01	18.49	26.35

Note: All LLM-based results in Table 4 are reported as the mean RMSE across multiple evaluation runs, with standard deviation reflecting the variability introduced by LLM stochasticity (temperature = 0.2), thereby ensuring a robust comparison to the ARDC baseline.

**Table 5:** Few-shot CoT LLM reproducibility evaluation metrics.

Azure OpenAI Models	Mean	STD	MAD	CV
gpt-4o	55.27	14.64	13.25	0.26
gpt-4o -mini	54.35	16.12	12.83	0.30
gpt-4-turbo	52.37	19.27	15.38	0.37
gpt-4	52.00	18.63	15.30	0.36
gpt-3.5-turbo	52.90	17.04	12.19	0.32

## Data Availability

Data sources and GitHub repository can be found in the Supporting Information file

## Environmental Implication

Per- and Polyfluoroalkyl Substances (PFAS) pose persistent environmental health risk, yet fragmented data sharing has limited monitoring and therefore policy. We present FAIR+Environmental, a large language model (LLM)-based framework that evaluates PFAS dataset quality and integration alongside FAIR principles. U.S. monitoring remains uneven because many datasets lack standardized metadata reporting and soil datasets especially lack widespread location coverage. Nearly 34 % of drinking water samples exceeded the PFOA maximum contaminant level (MCL).
