# Supplementary material for: Enhancing PFAS data integrity: An LLM-based FAIR+Environmental principle for improved evaluation of environmental contaminants and related constituent databases
Source: J Hazard Mater Adv. Author manuscript; Available in PMC 2026 Sep 10. (PMC13557099; doi:10.1016/j.hazadv.2026.101103)
Supplement: Supplementary file 1 [file NIHMS2183421-supplement-MMC2.docx]

**Enhancing PFAS Data Integrity: an LLM-Based FAIR+Environmental Principle for Improved Evaluation of Environmental Contaminants and Related Constituent Databases**

**Jialin Dong^1^, Sean D. Young^2,3^, Zixin Hu^1^, and Christopher I Olivares^1^**

^1^ Department of Civil and Environmental Engineering, University of California, Irvine, CA

^2^ Department of Informatics, University of California, Irvine, CA

^3^ Department of Emergency Medicine, University of California, Irvine, CA

*	Correspondence: [chris.olivares@uci.edu](mailto:chris.olivares@uci.edu)

Table of Contents

[**Table S1.** Summary of PFAS Datasets	4](#_Toc215008127)

[**Text S1.** Detailed Methods for LLM-Based FAIR Assessment and Rule-Based Score Extraction	22](#_Toc215008128)

[**Text S2.** Relationship Between PFAS Databases and Research Publications	24](#_Toc215008129)

[**Figure S1.** The number of current published PFAS relevant scientific papers for each state	25](#_Toc215008130)

[**Table S2.** Model-Specific Robustness in Markdown Output Generation and Parsing	25](#_Toc215008131)

[**Table S3.** Summary of FAIR Scores for PFAS Data Across Different Environmental Matrices	25](#_Toc215008132)

[**Table S4.** PFAS databases FAIR scores results evaluated by few-shot CoT LLM (ChatGPT 4o)	26](#_Toc215008133)

[**Table S5.** PFAS databases FAIR scores statistical analysis results evaluated by few-shot CoT LLM (ChatGPT 4o)	32](#_Toc215008134)

[**Figure S2.** Distribution of FAIR Principles Scores by State.	34](#_Toc215008135)

[**Figure S3.** F vs A Scores by different database (FAIR Size Weighted)	35](#_Toc215008136)

[**Figure S4.** I vs R Scores by different database (FAIR Size Weighted)	35](#_Toc215008137)

[**Text S3.** Groundwater PFOA Concentration Data Distribution Summary	35](#_Toc215008138)

[**Figure S5.** Groundwater PFOA Log Concentration by state from non PFAS contaminated sources	36](#_Toc215008139)

[**Figure S6.** Groundwater PFOA Log Concentration by state from known PFAS contaminated sources	36](#_Toc215008140)

[**Table S6.** Groundwater PFOA trend analysis for each state based on monthly medians of PFOA concentrations from non-PFAS-contaminated sources	37](#_Toc215008141)

[**Figure S7.** Surface water PFOA Concentration (ng/L) distribution by data source (log-scaled). The vertical blue line separates single-state (left) and multi-state (right) data sources. Sources and sample counts (years): Florida Department of Environmental Protection (DEP) (35, 2020–2022), Georgia Environmental Protection Division (EPD) (37, 2016), EPA Unregulated Contaminant Monitoring Rule 3 (UCMR3) (2269, 2013–2016), South Carolina Department of Health and Environmental Control (DHEC) (91, 2017–2020), University of Colorado (8, 2018–2019), Evaluation of Kentucky Community Drinking Water (80, 2019), Virginia Department of Health (DOH) Office of Drinking Water (11 locations, 2022), Texas Department of State Health Services (DSHS) (7, 2021), MDE (3, 2021), Maine.gov (70 locations, 2013–2023), EPA Unregulated Contaminant Monitoring Rule 5 (UCMR5) (2147 locations, 2023–2024).	39](#_Toc215008142)

[**Text S4.** Surface water PFOA Concentration Data Distribution Summary	39](#_Toc215008143)

[**Figure S8.** Surface water PFOA Log Concentration by state including all PFAS sources	40](#_Toc215008144)

[**Figure S9.** Surface water PFOA Log Concentration by state from non PFAS contaminated source	41](#_Toc215008145)

[**Table S7.** Surface water PFOA trend analysis for each state based on monthly medians of PFOA concentrations from non-PFAS-contaminated sources	41](#_Toc215008146)

[**Text S5.** Drinking water PFOA Concentration Data Distribution Summary	42](#_Toc215008147)

[**Figure S10.** Drinking water PFOA Log Concentration by state from non PFAS contaminated source	43](#_Toc215008148)

[**Figure S11.** Drinking water PFOA Log Concentration by state from PFAS contaminated source	43](#_Toc215008149)

[**Figure S12.** Drinking Water PFOA Concentration Distribution by State (All data). The horizontal blue dashed line represents a concentration of 4 ng/L.	44](#_Toc215008150)

[**Table S8.** Drinking water PFOA trend analysis for each state based on monthly medians of PFOA concentrations from all sources data	44](#_Toc215008151)

[**Text S6.** Soil PFOA Concentration Data Distribution Summary	46](#_Toc215008152)

[**Figure S13.** Soil PFOA Log Concentration by state and year from PFAS contaminated source	47](#_Toc215008153)

## **Table S1.** Summary of PFAS Datasets

| Media | Data Source | States Covered | Website | Data |
| --- | --- | --- | --- | --- |
| Groundwater | EWG | Alaska, Texas, California, Montana, New Mexico, Arizona, Nevada, Colorado, Oregon, Wyoming, Michigan, Minnesota, Utah, Idaho, Kansas, Nebraska, South Dakota, Washington, North Dakota, Oklahoma, Missouri, Florida, Wisconsin, Georgia, Illinois, Iowa, New York, North Carolina, Arkansas, Alabama, Louisiana, Mississippi, Pennsylvania, Virginia, Tennessee, Kentucky, Indiana, Maine, South Carolina, West Virginia, Maryland, Hawaii, Massachusetts, Vermont, New Hampshire, New Jersey, Connecticut, Delaware, Rhode Island, Ohio | <https://www.ewg.org/interactive-maps/pfas_contamination/map/> | <https://docs.google.com/spreadsheets/d/1dvNMDK6n3nw2RgZadVGaZk2JweW0EiukIelDLsiefa4/edit?usp=drive_link> |
| Groundwater | UCMR3 | Alaska, Texas, California, Montana, New Mexico, Arizona, Nevada, Oregon, Wyoming, Michigan, Minnesota, Utah, Kansas, Nebraska, Washington, North Dakota, Oklahoma, Missouri, Florida, Georgia, Illinois, New York, North Carolina, Alabama, Pennsylvania, Ohio, Tennessee, Kentucky, Maine, South Carolina, Maryland, Hawaii, Massachusetts, Vermont, New Hampshire, New Jersey, Connecticut, Delaware, Rhode Island, Louisiana | <https://www.epa.gov/dwucmr/occurrence-data-unregulated-contaminant-monitoring-rule#3> | <https://www.epa.gov/system/files/other-files/2024-04/ucmr3-occurrence-data.zip> |
| Groundwater | PPL | Alaska, Texas, California, Montana, New Mexico, Arizona, Nevada, Colorado, Oregon, Wyoming, Michigan, Minnesota, Utah, Idaho, Kansas, Nebraska, South Dakota, Washington, North Dakota, Oklahoma, Missouri, Florida, Wisconsin, Georgia, Illinois, Iowa, New York, North Carolina, Arkansas, Alabama, Mississippi, Pennsylvania, Ohio, Tennessee, Kentucky, Indiana, Maine, South Carolina, West Virginia, Maryland, Hawaii, Massachusetts, Vermont, New Hampshire, New Jersey, Connecticut, Delaware, Rhode Island, Louisiana, Virginia | <https://experience.arcgis.com/experience/12412ab41b3141598e0bb48523a7c940/page/Page-1/?views=Known-Contamination> | <https://docs.google.com/spreadsheets/d/1-LSXJbA_3M20n76y-FK0YLd1fml1uzJsoAkSWr2bz0A/edit#gid=2141223452> |
| Groundwater | UCMR5 | Alaska, Texas, California, Montana, New Mexico, Arizona, Nevada, Colorado, Oregon, Wyoming, Michigan, Minnesota, Utah, Idaho, Kansas, Nebraska, South Dakota, Washington, North Dakota, Oklahoma, Missouri, Florida, Wisconsin, Georgia, Iowa, New York, North Carolina, Arkansas, Alabama, Louisiana, Mississippi, Pennsylvania, Virginia, Tennessee, Kentucky, Indiana, Maine, South Carolina, West Virginia, Maryland, Hawaii, Massachusetts, Vermont, New Hampshire, New Jersey, Connecticut, Delaware, Rhode Island, Ohio, Illinois | <https://www.epa.gov/dwucmr/occurrence-data-unregulated-contaminant-monitoring-rule#3> | <https://www.epa.gov/system/files/other-files/2023-08/ucmr5-occurrence-data.zip> |
| Groundwater | Alaska DEC | Alaska | <https://dec.alaska.gov/spar/csp/pfas/responses/> | <https://dot.alaska.gov/airportwater/fairbanks/> |
| Groundwater | GAMA/DDW | California | <https://data.ca.gov/dataset/ground-water-water-quality-results/resource/c7cd50a2-2a68-44a1-8d40-4e767f363964> | <https://data.ca.gov/dataset/81d1347c-891d-4f09-abc5-6eeb521b55d2/resource/c7cd50a2-2a68-44a1-8d40-4e767f363964/download/pfas.csv> |
| Groundwater | GeoTracker | California | <https://geotracker.waterboards.ca.gov/> | <https://geotracker.waterboards.ca.gov/datadownload> |
| Groundwater | USGS | California, Virginia, West Virginia, Massachusetts | <https://www.sciencebase.gov/catalog/item/60db3fe9d34e596d2ba5c8f7> | <https://docs.google.com/spreadsheets/d/17B4eZfMXErwC8Gc2JlCzIdJinYFMmzGX/edit?usp=drive_link&ouid=110505850797239939089&rtpof=true&sd=true> |
| Groundwater | NMED | New Mexico | <https://www.env.nm.gov/public-record-request/> | <https://docs.google.com/spreadsheets/d/1J9ypRjNSFVYEJWWuKQKUp7aiKzZ8DmW_/edit?gid=1356189441#gid=1356189441> |
| Groundwater | ADEQ | Arizona | <https://www.azdeq.gov/LAFB_PFAS> | <https://drive.google.com/file/d/1x3ESxbv5Wj20Zp6BQt17eU-7RQGvrw38/view?usp=sharing> |
| Groundwater | CDPHE | Colorado | <https://cohealthviz.dphe.state.co.us/t/EnvironmentalEpidemiologyPublic/views/PFAS_results_DRAFT/Download?%3Adisplay_count=n&%3Aembed=y&%3AisGuestRedirect&%3AisGuestRedirectFromVizportal=y&%3Aorigin=viz_share_link&%3AshowAppBanner=false&%3AshowVizHome=n> | <https://drive.google.com/file/d/1Am-1b3pkLqNPrBDRMdSE9R6LPbFBLtCf/view> |
| Groundwater | (Jackson Hole airport) | Wyoming | <https://jhair.wpenginepowered.com/wp-content/uploads/2022/12/FINAL-JAC-Cummulative-airport-well-info-for-website-12-2022.pdf> | <https://www.jacksonholeairport.com/wp-content/uploads/2020/11/JAC-Residential-well-test-areas-Phase-123.pdf> |
| Groundwater | EGLE | Michigan | <https://gis-egle.hub.arcgis.com/search?q=pfas> | <https://docs.google.com/spreadsheets/d/1FQffi-tlex5eUJhFtcerzkTdSjnxXMc8HZtfKicZic0/edit?usp=sharing> |
| Groundwater | MPCA | Minnesota | <https://mdh.maps.arcgis.com/apps/MapSeries/index.html?appid=63515695237f425ea7120d1aac1fd09a> | <https://docs.google.com/spreadsheets/d/1NW4qR-sicy8VVydI0NrWCvSF8jtPW4ny/edit?usp=drive_link&ouid=111590302547849128417&rtpof=true&sd=true> |
| Groundwater | Utah DEQ | Utah | <https://utahdeq.maps.arcgis.com/apps/webappviewer/index.html?id=406524c54f4845258ffa6912f06e23e4> | <https://docs.google.com/spreadsheets/d/1rk0jZ0P7swH0rczb5K8YmvxaU6OOvEuG/edit?usp=drive_link&ouid=110505850797239939089&rtpof=true&sd=true> |
| Groundwater | North Carolina DEQ | North Carolina | <https://www.deq.nc.gov/dwr-gwr-lcfr-pfas-data-2019-2021> | <https://www.deq.nc.gov/dwr-gwr-lcfr-pfas-data-2019-2021/open> |
| Groundwater | IDEQ | Idaho | <https://www.deq.idaho.gov/public-records-request/> | [Idaho_PRR - PFAS Sampling with Date and GPS Coordinates (GW).xlsx](https://docs.google.com/spreadsheets/d/1Eq7y4uyLTt-SZKCjdApYs4wSpIIJ661I/edit?usp=drive_link&ouid=110505850797239939089&rtpof=true&sd=true) |
| Groundwater | Seattle Public Utilities | Washington | <https://www.seattle.gov/utilities/your-services/water/water-quality/quality-concerns/pfas> | <https://drive.google.com/file/d/1k94Gle_GeflAPjJ7ijx-qHmhSERLWS8D/view?usp=drive_link> |
| Groundwater | NDDEQ | North Dakota | <https://deq.nd.gov/MF/PFAS/> | <https://deq.nd.gov/Publications/MF/PFAS_Report.pdf?v=2> |
| Groundwater | Missouri PFAS Viewer | Missouri | <https://modnr.maps.arcgis.com/apps/webappviewer/index.html?id=386c71927569476ebd2d0e6910424d17> | <https://drive.google.com/file/d/15lFSKdXHXBva9-W9S8CQY-sAHupDf1A1/view?usp=drive_link> |
| Groundwater | fight4zero | Florida | <https://www.fight4zero.org/pfasmap> |  |
| Groundwater | ERIC | Florida | <https://geodata.dep.state.fl.us/datasets/FDEP::eric-pfas-sites-1/about> |  |
| Groundwater | EPD | Georgia | <https://gaepd.maps.arcgis.com/apps/MapSeries/index.html?appid=e8f2c6a51c1c41088002350f1eabe598> | <https://docs.google.com/spreadsheets/d/19ft5AL_gt4brjPjcQp2FYOLfxuVhASgb/edit?usp=drive_link&ouid=111590302547849128417&rtpof=true&sd=true> |
| Groundwater | ERISIllinois EPA's PFAS Sampling Network | Illinois | <https://www.arcgis.com/apps/View/index.html?appid=1efa49761b194e3190c69404b12cf1e5> |  |
| Groundwater | DNR | Iowa | <https://experience.arcgis.com/experience/b04e0e828a974e6e8962e47895ebb520> | <https://docs.google.com/document/d/1NUVWZOxbEfJxR9Jrs7WFYZyJ6sc0_V_m/edit> |
| Groundwater | PFAS in NYS | New York | <https://storymaps.arcgis.com/stories/f3b905cc113c4b94911283716a7b583d> |  |
| Groundwater | Arkansas Department of Energy and Environment | Arkansas | <https://www.adeq.state.ar.us/techsvs/env_multi_lab/water_quality_station.aspx#Display> | <https://www.adeq.state.ar.us/downloads/WebDatabases/TechnicalServices/WQARWebLIMS/WQARWebLIMS_web.zip> |
| Groundwater | ADEM | Alabama | <https://adem.alabama.gov/programs/water/drinkingwater/pfaspage.cnt> | <https://drive.google.com/file/d/1AiADGYgNjgDaKQFOInd84zzjixJaHrFJ/view?usp=drive_link> |
| Groundwater | Ohio Environmental Protection Agency | Ohio | <https://data-oepa.opendata.arcgis.com/datasets/oepa::pfas-sampling-results/about> | <https://docs.google.com/spreadsheets/d/1zqIk2t5XZDUh_x0IFnTTTOBS8UQ5siUEvDRXdjH__0c/edit?usp=drive_link> |
| Groundwater | Virginia Department of Health Office of Drinking Water | Virginia | <https://www.vdh.virginia.gov/drinking-water/> | <https://docs.google.com/document/d/1S1sQ6lCDY-LRr0MIiL4DY1Omr1GkZuVG/edit?usp=drive_link&ouid=110505850797239939089&rtpof=true&sd=true> |
| Groundwater | TDEC | Tennessee | <https://www.tn.gov/environment/policy/pfas/tdec-sampling-for-pfas.html> | <https://drive.google.com/file/d/1lRLdV-Wfs0gjS3Ruvs0t9lIPWP_fG9q4/view?usp=drive_link> |
| Groundwater | Evaluation of Kentucky Community Drinking Water | Kentucky | <https://eec.ky.gov/pages/search.aspx?terms=pfas&affiliateId=EEC> | [https://eec.ky.gov/Documents%20for%20URLs/PFAS%20Drinking%20Water%20Report%20Final.pdf](https://eec.ky.gov/pages/search.aspx?terms=pfas&affiliateId=EEC) |
| Groundwater | Maine.gov | Maine | <https://www.maine.gov/dep/spills/topics/pfas/> | <https://docs.google.com/spreadsheets/d/1mFDOk8dfsXZ7f2Rp8crF1o9JuxVUMoXL/edit?usp=drive_link&ouid=111590302547849128417&rtpof=true&sd=true> |
| Groundwater | Ambient Surface Water PFAS Project | South Carolina | <https://gis.dhec.sc.gov/gisportal/apps/webappviewer/index.html?id=162b8d1e7fdf459db7ff6b251671651f> | <https://gis.dhec.sc.gov/Water_Web_Docs/PFAS/Ambient%20Surface%20Water%20Project%20-%20Surface%20Water%20Data.xlsx> |
| Groundwater | GOV | South Carolina | <https://scdhec.gov/environment/polyfluoroalkyl-substances-pfas/pfas-bureau-water/pfas-drinking-water-sampling-results> | <https://scdhec.gov/sites/default/files/media/document/T9.pdf> |
| Groundwater | MDE | Maryland | <https://mde.maryland.gov/PublicHealth/Pages/PFAS-Landing-Page.aspx> | <https://mde.maryland.gov/programs/water/water_supply/Documents/Phase3PFASResultsTable.xlsx> |
| Groundwater | Safe Waters | Hawaii | <https://jbphh-safewaters.org/public/framework/bannerhtml.aspx?idhtml=10773&banner=jbphh_environmental.png&title=PFAS%20Sampling&Domain=env.jbphh-safewaters.org&idMenu=92559&ddlDSN=SYSTM&DSN=SYSTM> | <https://jbphh-safewaters.org/public/PFAS_Groundwater_Results_January_2023.pdf> |
| Groundwater | NHDES | New Hampshire | <https://nhdes.maps.arcgis.com/apps/View/index.html?appid=66770bef141c43a98a445c54a17720e2&extent=-73.5743,42.5413,-69.6852,45.4489> |  |
| Groundwater | NJDEP | New Jersey | <https://dep.nj.gov/contact/> | <https://docs.google.com/spreadsheets/d/1Pa4TeqKaEAzllhBWLIRY5lnG6I2mJDd2/edit?usp=drive_link&ouid=110505850797239939089&rtpof=true&sd=true> |
| Groundwater | Massachusetts Sierra Club | Rhode Island | <https://www.sierraclub.org/sites/default/files/sce/massachusetts-chapter/2021/PFAS-res/PFAS-RI-water-2022-01.pdf> | <https://www.sierraclub.org/sites/default/files/sce/massachusetts-chapter/2021/PFAS-res/PFAS-RI-water-2022-01.pdf> |
| Drinking Water | Alaska DEC | Alaska | <https://dec.alaska.gov/spar/csp/pfas/sample-results/> | <https://dec.alaska.gov/media/wewnuyon/anchorage-aia-february-2023.pdf> |
| Drinking Water | DoD | Alaska, Texas, California, Montana, New Mexico, Arizona, Colorado, Oregon, Michigan, Minnesota, Kansas, Washington, Oklahoma, Florida, Illinois, New York, North Carolina, Arkansas, Ohio, Virginia, South Carolina, Hawaii, New Hampshire, New Jersey, Delaware, Rhode Island, Massachusetts, South Dakota, Georgia, Pennsylvania, Tennessee, Maine, Maryland | <https://www.acq.osd.mil/eie/eer/ecc/pfas/map/pfasmap.html> | <https://www.acq.osd.mil/eie/eer/ecc/pfas/map/docs/final/Mather%20AFB%2020221017%20-%2020221027.pdf> |
| Drinking Water | EWG | Alaska, Texas, California, Montana, Arizona, Nevada, Colorado, Oregon, Wyoming, Michigan, Minnesota, Utah, Idaho, Kansas, Washington, North Dakota, Missouri, Florida, Wisconsin, Illinois, Iowa, New York, North Carolina, Arkansas, Alabama, Louisiana, Mississippi, Ohio, Kentucky, Indiana, South Carolina, West Virginia, Hawaii, Massachusetts, New Hampshire, New Jersey, Connecticut, Delaware, Rhode Island, Nebraska, South Dakota, Georgia, Pennsylvania, Virginia, Tennessee, Maine, Maryland | <https://www.ewg.org/interactive-maps/pfas_contamination/map/> |  |
| Drinking Water | PPL | Alaska, Texas, California, Montana, New Mexico, Arizona, Oregon, Wyoming, Michigan, Minnesota, Utah, Kansas, South Dakota, Washington, Oklahoma, Missouri, Florida, Wisconsin, Illinois, New York, North Carolina, Arkansas, Louisiana, Ohio, Indiana, Massachusetts, Vermont, New Jersey, Connecticut, Rhode Island, Nebraska, North Dakota, Mississippi, New Hampshire, Pennsylvania, Maine, Maryland | <https://experience.arcgis.com/experience/12412ab41b3141598e0bb48523a7c940/page/Page-1/?views=Known-Contamination> | <https://docs.google.com/spreadsheets/d/1-LSXJbA_3M20n76y-FK0YLd1fml1uzJsoAkSWr2bz0A/edit#gid=2141223452> |
| Drinking Water | CA.gov | California | <https://geotracker.waterboards.ca.gov/map/pfas_map> |  |
| Drinking Water | GAMA | California | <https://data.ca.gov/dataset/ground-water-water-quality-results/resource/be2d189b-dcb7-4c1c-b881-a52b278cf0a7> | <https://data.ca.gov/dataset/81d1347c-891d-4f09-abc5-6eeb521b55d2/resource/be2d189b-dcb7-4c1c-b881-a52b278cf0a7/download/ddw2015-2020_2023-10-02.csv> |
| Drinking Water | gispublic | California | <https://experience.arcgis.com/experience/12412ab41b3141598e0bb48523a7c940/page/Page-1/?views=Known-Contamination> | <https://docs.google.com/spreadsheets/d/1-LSXJbA_3M20n76y-FK0YLd1fml1uzJsoAkSWr2bz0A/edit#gid=2141223452> |
| Drinking Water | NMED | New Mexico | <https://www.env.nm.gov/pfas/data/> | <https://www.env.nm.gov/wp-content/uploads/sites/21/2019/04/Alamogordo-Holloman-PFAS-sample-results.pdf> |
| Drinking Water | CDPHE | Colorado | <https://cdphe.maps.arcgis.com/apps/MapSeries/index.html?appid=0fd782d4fe2f49ed967b682e10573834> | <https://drive.google.com/file/d/1AiSEOrGgWi1U4owD1hOEKA2q1xTy8NOF/view> |
| Drinking Water | OHA | Oregon | <https://www.oregon.gov/oha/PH/HEALTHYENVIRONMENTS/DRINKINGWATER/OPERATIONS/Pages/PFAS.aspx> | <https://www.oregon.gov/oha/PH/HEALTHYENVIRONMENTS/DRINKINGWATER/OPERATIONS/Documents/UCMR3-PFAS-only-data-OR.xlsx> |
| Drinking Water | DOH | Washington | <https://doh.wa.gov/data-and-statistical-reports/washington-tracking-network-wtn/pfas/dashboard> | <https://docs.google.com/spreadsheets/d/1bExlSY2EQO8xf2aKts3-Mbevq4P9WfEh/edit?usp=drive_link&ouid=111590302547849128417&rtpof=true&sd=true> |
| Drinking Water | NDDEQ | North Dakota | <https://deq.nd.gov/MF/PFAS/> | <https://deq.nd.gov/Publications/MF/PFAS_Report_2020.pdf?v=1> |
| Drinking Water | fight4zero | Florida | <https://www.fight4zero.org/pfasmap> |  |
| Drinking Water | Wisconsin Department of Natural Resource | Wisconsin | <https://dnr.wi.gov/dwsviewer/> | <https://apps.dnr.wi.gov/dwsportalpub/ContamResult/Search> |
| Drinking Water | Village of East Troy | Wisconsin | <https://easttroywi.gov/vertical/sites/%7BF9A16C36-7BFC-4D96-8E31-D88549B56F12%7D/> | <https://easttroywi.gov/vertical/sites/%7BF9A16C36-7BFC-4D96-8E31-D88549B56F12%7D/uploads/Village_of_East_Troy_PFAS_Sampling_Results_2022.pdf> |
| Drinking Water | EPD | Georgia | <https://epd.georgia.gov/pfoa-and-pfos-information> | <https://gaepd.maps.arcgis.com/apps/MapSeries/index.html?appid=e8f2c6a51c1c41088002350f1eabe598> |
| Drinking Water | Illinois EPA | Illinois | <https://illinois-epa.maps.arcgis.com/apps/dashboards/bd611162a7f74cfe88b6928c926416c3> | <https://epa.illinois.gov/topics/water-quality/pfas/pfas-statewide-investigation-network.html> |
| Drinking Water | PFAS IN NYS | New York | <https://storymaps.arcgis.com/stories/f3b905cc113c4b94911283716a7b583d> |  |
| Drinking Water | DEP | Pennsylvania | <https://www.dep.pa.gov/Citizens/My-Water/drinking_water/PFAS/Pages/default.aspx> | <https://files.dep.state.pa.us/Water/DrinkingWater/Perfluorinated%20Chemicals/SamplingResults/PFAS_Sampling_Final_Results_May_2021.pdf> |
| Drinking Water | VIRGINIA DEPARTMENT OF HEALTH | Virginia | <https://www.deq.virginia.gov/get-involved/the-environment-you/per-and-polyfluoroalkyl-substances-pfas> | <https://docs.google.com/spreadsheets/d/1A41x30C88Fb_8qDEH6ffirQoFyN0JdiQ/edit?usp=sharing&ouid=110505850797239939089&rtpof=true&sd=true> |
| Drinking Water | TDEC | Tennessee | <https://www.tn.gov/environment/policy/pfas.html> | <https://experience.arcgis.com/experience/0e7d9916f6b94f92b0cc9c07d30abe69> |
| Drinking Water | Indiana Department of Environmental Management | Indiana | <https://www.in.gov/idem/resources/nonrule-policies/per-and-polyfluoroalkyl-substances-pfas/> | <https://www.in.gov/idem/files/pfas_results_1.xlsx> |
| Drinking Water | Maine DWP | Maine | <https://www.maine.gov/dhhs/mecdc/environmental-health/dwp/pws/pfas.shtml#results> | <https://www.maine.gov/dhhs/mecdc/environmental-health/dwp/cet/documents/pfasResults.pdf> |
| Drinking Water | PPL PFAS Drinking Water Sampling Results | South Carolina | <https://scdhec.gov/environment/polyfluoroalkyl-substances-pfas/pfas-bureau-water/pfas-drinking-water-sampling-results> | <https://scdhec.gov/sites/default/files/media/document/T13%20Other%20PFAS%20by%20537.1.pdf> |
| Drinking Water | MDE | Maryland | <https://mde.maryland.gov/programs/Water/water_supply/Pages/PFAS_Home.aspx> | <https://mde.maryland.gov/programs/water/water_supply/Documents/Phase4PFASResulstTable.xlsx> |
| Drinking Water | DOH report | Hawaii | <https://health.hawaii.gov/heer/environmental-health/highlighted-projects/pfas/> | <https://health.hawaii.gov/news/submenu/pfas-detected-in-maui-business-park-system/> |
| Drinking Water | MassGOV | Massachusetts | <https://eeaonline.eea.state.ma.us/portal#!/search/drinking-water> |  |
| Drinking Water | usgs | Massachusetts | <https://www.usgs.gov/publications/and-polyfluoroalkyl-substances-pfas-united-states-tapwater-comparison-underserved> | <https://ars.els-cdn.com/content/image/1-s2.0-S0160412023003069-mmc2.xlsx> |
| Drinking Water | Commenwealth of Mass | Massachusetts | <https://malegislature.gov/Bills/192/HD5054> | <https://malegislature.gov/Bills/192/HD5054.pdf> |
| Drinking Water | DWGPD | Vermont | <https://dec.vermont.gov/water/drinking-water/pfas> | <https://anrweb.vt.gov/DEC/DWGWP/license.aspx?Report=PFASData> |
| Drinking Water | State of Rhode Island Department of Health | Rhode Island | <https://health.ri.gov/data/pfas/> | <https://health.ri.gov/publications/reports/Update-on-PFAS-Act-Compliance.pdf> |
| Surface Water | UCMR5 | Alaska, Texas, California, Colorado, Oregon, Michigan, Minnesota, Idaho, Washington, Oklahoma, Missouri, Florida, Wisconsin, Georgia, Iowa, New York, Arkansas, Alabama, Louisiana, Mississippi, Pennsylvania, Tennessee, Kentucky, Indiana, Maine, South Carolina, West Virginia, Maryland, Hawaii, Massachusetts, Vermont, New Jersey, Connecticut, Rhode Island, Montana, Nevada, Wyoming, Utah, Nebraska, North Dakota, Illinois, North Carolina, New Hampshire, Delaware, Virginia | <https://www.epa.gov/dwucmr/occurrence-data-unregulated-contaminant-monitoring-rule#3> | <https://www.epa.gov/system/files/other-files/2023-08/ucmr5-occurrence-data.zip> |
| Surface Water | PPL | Alaska, California, Colorado, Michigan, Idaho, South Dakota, Washington, Wisconsin, Georgia, New York, North Carolina, Tennessee, Maine, Maryland, New Jersey, Connecticut | <https://experience.arcgis.com/experience/12412ab41b3141598e0bb48523a7c940/page/Page-1/?views=Known-Contamination> | <https://docs.google.com/spreadsheets/d/1-LSXJbA_3M20n76y-FK0YLd1fml1uzJsoAkSWr2bz0A/edit#gid=2141223452> |
| Surface Water | UCMR3 | Texas, California, Montana, Nevada, Oregon, Wyoming, Michigan, Minnesota, Utah, Nebraska, Washington, North Dakota, Missouri, Wisconsin, Georgia, Illinois, New York, North Carolina, Alabama, Louisiana, Pennsylvania, Tennessee, Kentucky, Maine, South Carolina, Maryland, Hawaii, Massachusetts, Vermont, New Hampshire, New Jersey, Connecticut, Delaware, Rhode Island | <https://www.epa.gov/dwucmr/occurrence-data-unregulated-contaminant-monitoring-rule#3> | <https://www.epa.gov/system/files/other-files/2024-04/ucmr3-occurrence-data.zip> |
| Surface Water | DSHS | Texas | <https://www.dshs.texas.gov/seafood-aquatic-life-group/information-on-consumption-advisories-possession-bans-rescinded-orders-seafood-aquatic-life/fishing-advisories-bans-faqs> | <https://www.dshs.texas.gov/sites/default/files/seafood/PDF2/Risk-Characterization/Lower%20Leon%20Creek%20Risk%20Characterization%20Addendum%202022.pdf> |
| Surface Water | SFEI | California | <https://www.sfei.org/projects/pfas#:~:text=A%20recent%20analysis%20of%20PFAS,22%20sites%20in%20the%20Bay.> | <https://www.sfei.org/sites/default/files/biblio_files/PFASinBayWater2022_Final_0.pdf> |
| Surface Water | UCMR3，UCMR5 | New Mexico, Arizona, Kansas | <https://www.epa.gov/dwucmr/occurrence-data-unregulated-contaminant-monitoring-rule#3> | <https://www.epa.gov/system/files/other-files/2024-04/ucmr3-occurrence-data.zip> |
| Surface Water | CDPHE | Colorado | <https://cdphe.maps.arcgis.com/apps/MapSeries/index.html?appid=0fd782d4fe2f49ed967b682e10573834> | <https://cdphe.maps.arcgis.com/apps/MapSeries/index.html?appid=0fd782d4fe2f49ed967b682e10573834> |
| Surface Water | DCD | Colorado | <https://data.mendeley.com/datasets/dym93kv36x/3> | <https://prod-dcd-datasets-cache-zipfiles.s3.eu-west-1.amazonaws.com/dym93kv36x-3.zip> |
| Surface Water | University of Colorado at Colorado Springs | Colorado | <https://data.mendeley.com/datasets/dym93kv36x/3> | <https://prod-dcd-datasets-cache-zipfiles.s3.eu-west-1.amazonaws.com/dym93kv36x-3.zip> |
| Surface Water | EGLE | Michigan | <https://gis-egle.hub.arcgis.com/maps/391cca4f364845829abcd5a92093c631/about> | <https://www.arcgis.com/sharing/rest/content/items/391cca4f364845829abcd5a92093c631/info/metadata/metadata.xml?format=default&output=html> |
| Surface Water | UCMR3.UCMR5 | South Dakota | <https://www.epa.gov/dwucmr/occurrence-data-unregulated-contaminant-monitoring-rule#3> | <https://www.epa.gov/system/files/other-files/2023-08/ucmr5-occurrence-data.zip> |
| Surface Water | NDDEQ | North Dakota | <https://deq.nd.gov/MF/PFAS/> | <https://deq.nd.gov/Publications/MF/PFASRedRiver2022_ResultsSummary.pdf?v=1> |
| Surface Water | Missouri PFAS Viewer | Missouri | <https://modnr.maps.arcgis.com/apps/webappviewer/index.html?id=386c71927569476ebd2d0e6910424d17> | <https://drive.google.com/drive/folders/1MDFDlH99ZjEpvQJxJrz52HoyA-6DTi-G?usp=share_link> |
| Surface Water | DEQ | Florida, North Carolina, utah, | <https://ncdenr.maps.arcgis.com/apps/dashboards/d20ef253863b432db0aceaa510d7feda> |  |
| Surface Water | Florida Department of Environmental Protection | Florida, Georgia | <https://wwals.net/2022/10/18/forever-chemicals-contaminate-withlacoochee-river-in-georgia-and-florida-2022-10-18/> | <https://www.wwals.net/pictures/2022-10-18--wka-pfas-report/georgia-test-results.html> |
| Surface Water | EPD | Georgia | <https://epd.georgia.gov/pfoa-and-pfos-information> | <https://gaepd.maps.arcgis.com/apps/MapSeries/index.html?appid=e8f2c6a51c1c41088002350f1eabe598> |
| Surface Water | Illinois EPA's PFAS Sampling Network | Illinois | <https://illinois-epa.maps.arcgis.com/apps/dashboards/bd611162a7f74cfe88b6928c926416c3> |  |
| Surface Water | UCMR5 DEQ UCMR3 | Ohio | <https://data-oepa.opendata.arcgis.com/datasets/oepa::pfas-sampling-results/about> | <https://data-oepa.opendata.arcgis.com/datasets/pfas-sampling-results/explore?showTable=true> |
| Surface Water | WRRC | Virginia | <https://www.vwrrc.vt.edu/> | <https://www.vwrrc.vt.edu/wp-content/uploads/2022/08/SR-66_2022_PFAS-Monitoring-Options-to-Evaluate-Virginias-Surface-Waters.pdf> |
| Surface Water | Virginia Department of Health Office of Drinking Water | Virginia | <https://www.deq.virginia.gov/get-involved/the-environment-you/per-and-polyfluoroalkyl-substances-pfas> | <https://docs.google.com/spreadsheets/d/1A41x30C88Fb_8qDEH6ffirQoFyN0JdiQ/edit?usp=sharing&ouid=110505850797239939089&rtpof=true&sd=true> |
| Surface Water | TDEC | Tennessee | <https://www.tn.gov/environment/policy/pfas.html> | <https://experience.arcgis.com/experience/0e7d9916f6b94f92b0cc9c07d30abe69> |
| Surface Water | Evaluation of Kentucky Community Drinking Water | Kentucky | <https://eec.ky.gov/Environmental-Protection/Water/Protection/Pages/PFAS.aspx> | <https://eec.ky.gov/Documents%20for%20URLs/PFAS%20Drinking%20Water%20Report%20Final.pdf> |
| Surface Water | Maine.gov | Maine | <https://www.maine.gov/dep/spills/topics/pfas/> | <https://docs.google.com/spreadsheets/d/1mFDOk8dfsXZ7f2Rp8crF1o9JuxVUMoXL/edit?usp=drive_link&ouid=111590302547849128417&rtpof=true&sd=true> |
| Surface Water | PFAS Drinking Water Sampling Results | South Carolina | <https://scdhec.gov/environment/polyfluoroalkyl-substances-pfas/pfas-bureau-water/pfas-drinking-water-sampling-results> | <https://scdhec.gov/sites/default/files/media/document/Table%201.pdf> |
| Surface Water | MDE | Maryland | <https://mde.maryland.gov/PublicHealth/Pages/PFAS-Landing-Page.aspx> | <https://mde.maryland.gov/PublicHealth/Documents/Pisctaway_PFAS_Study_Final.pdf> |
| Surface Water | USGS | Massachusetts | <https://www.sciencebase.gov/catalog/item/5d7b9fe6e4b0c4f70d034212> | <https://www.sciencebase.gov/catalog/item/5d7b9fe6e4b0c4f70d034212> |
| Soil | PPL | Alaska, Texas, California, Washington, Missouri, Hawaii, Vermont | <https://experience.arcgis.com/experience/12412ab41b3141598e0bb48523a7c940/page/Page-1/?views=Known-Contamination> | <https://docs.google.com/spreadsheets/d/1-LSXJbA_3M20n76y-FK0YLd1fml1uzJsoAkSWr2bz0A/edit#gid=2141223452> |
| Soil | DSHS | Texas | <https://www.dshs.texas.gov/seafood-aquatic-life-group/information-on-consumption-advisories-possession-bans-rescinded-orders-seafood-aquatic-life/fishing-advisories-bans-faqs> | <https://www.dshs.texas.gov/sites/default/files/seafood/PDF2/Risk-Characterization/Lower%20Leon%20Creek%20Risk%20Characterization%20Addendum%202022.pdf> |
| Soil | PFAS Project Lab | California | <https://experience.arcgis.com/experience/12412ab41b3141598e0bb48523a7c940/page/Page-1/?views=Known-Contamination> | <https://docs.google.com/spreadsheets/d/1-LSXJbA_3M20n76y-FK0YLd1fml1uzJsoAkSWr2bz0A/edit#gid=2141223452> |
| Soil | NMED(email) | New Mexico | <https://www.env.nm.gov/pfas/data/> | [Rpt_2111381_Friedman_Recycling_Final_v1.pdf](https://drive.google.com/file/d/1PqdeKo_g00wKAAuVZV2l94HEqm2j_sdh/view?usp=sharing) |
| Soil | RWRD | Arizona | <https://nwbiosolids.org/> | <https://static1.squarespace.com/static/54806478e4b0dc44e1698e88/t/5fad464a2bb93a1ea26a8e1a/1605191256895/PepperEtAl2020-PFAS-in-Biosolids_A-Southern-Arizona-Case-Study.pdf> |
| Soil | CDPHE | Colorado | <https://cdphe.maps.arcgis.com/apps/MapSeries/index.html?appid=0fd782d4fe2f49ed967b682e10573834> | <https://cdphe.maps.arcgis.com/apps/MapSeries/index.html?appid=0fd782d4fe2f49ed967b682e10573834> |
| Soil | University of Colorado at Colorado Springs | Colorado | <https://data.mendeley.com/datasets/dym93kv36x/3> | <https://prod-dcd-datasets-cache-zipfiles.s3.eu-west-1.amazonaws.com/dym93kv36x-3.zip> |
| Soil | fight4zero | Florida | <https://www.fight4zero.org/pfasmap> |  |
| Soil | EWG | Florida | <https://www.ewg.org/interactive-maps/2020_suspected_industrial_discharges_of_pfas/map/> |  |
| Soil | Maine.gov/ PPL | Maine | <https://www.maine.gov/dep/spills/topics/pfas/> | <https://docs.google.com/spreadsheets/d/1mFDOk8dfsXZ7f2Rp8crF1o9JuxVUMoXL/edit?usp=drive_link&ouid=111590302547849128417&rtpof=true&sd=true> |
| Soil | DOH report | Hawaii | <https://jbphh-safewaters.org/public/framework/bannerhtml.aspx?idhtml=10773&banner=jbphh_environmental.png&title=PFAS%20Sampling&Domain=env.jbphh-safewaters.org&idMenu=92559&ddlDSN=SYSTM&DSN=SYSTM> | <https://jbphh-safewaters.org/public/PFAS_Soil_Sampling_Results_December_2022.pdf> |
| Soil | UNIVERSITY OF VERMONT | Vermont | <https://anrweb.vt.gov/> | <https://anrweb.vt.gov/PubDocs/DEC/PFOA/Soil-Background/PFAS-Background-Vermont-Shallow-Soils-03-24-19.pdf> |
| Soil | USGS | New Hampshire | [https://www.sciencebase.gov/catalog/item/61f43d6cd34e622189bbb0c4](https://www.google.com/url?q=https://www.sciencebase.gov/catalog/item/61f43d6cd34e622189bbb0c4&sa=D&source=editors&ust=1698781865319118&usg=AOvVaw3E9t5-bWZ2YoxU6UB56c6U) | <https://www.sciencebase.gov/catalog/item/61f43d6cd34e622189bbb0c4#:~:text=this%20item%20or-,download,-all%20files%20listed> |
| Soil | EPA | New Jersey | <https://catalog.data.gov/dataset/new-jersey-njdep-epa-ord-soil-pfas-study> | <https://pasteur.epa.gov/uploads/10.23719/1506012/Washington%20et%20al%20NJ%20Soil%20PFCAs%20For%20Online%20Storage.xlsx> |

## **Text S1.** Detailed Methods for LLM-Based FAIR Assessment and Rule-Based Score Extraction

This study developed a semi-automated FAIR assessment workflow that combines large language model (LLM) reasoning with a deterministic rule-based scoring and validation procedure. The workflow consists of four major components: (1) prompt construction and LLM evaluation, (2) structured markdown output generation, (3) rule-based extraction of numerical FAIR scores, and (4) validation and error-handling routines to ensure robustness across datasets.

LLM Prompting and Evaluation Framework

Each dataset was evaluated through a Python-based interface to Azure OpenAI’s GPT model. We constructed structured prompts that embed the FAIR rubric, including sub-criteria for Findability, Accessibility, Interoperability, and Reusability. Multiple prompting strategies (zero-shot, one-shot, and few-shot chain-of-thought) were tested to improve consistency, and the final workflow used few-shot CoT prompts with embedded worked examples. The LLM generated its assessments in standardized markdown table format to facilitate downstream parsing.

Preprocessing of Website Content for LLM Inputs

Because Azure OpenAI models do not possess native browsing or URL-fetching capabilities, all webpage content was retrieved through an explicit preprocessing step prior to LLM evaluation. For each dataset URL, a Python web-scraping routine (Requests + BeautifulSoup) extracted key metadata fields—including page title, meta description, license-related keywords, detected file formats, downloadable file links, and a snippet of visible text content (~2000 characters). This structured, scraped content was then passed directly into the LLM prompt, ensuring that all FAIR evaluations were based solely on reproducible webpage information rather than implicit model knowledge or hallucinated content. The full scraping script is included in the GitHub repository.

Standardized Markdown Output Format

The GPT model was instructed to return a four-row markdown table corresponding to the FAIR components (F, A, I, R), each containing a numerical score, along with a total FAIR score (maximum 42 points). Additional guidance was included to minimize formatting variability, reduce extraneous text, and ensure stable table structures suitable for automated parsing.

Rule-Based Parsing Logic for Score Extraction

A deterministic parsing layer was implemented to convert the LLM’s markdown output into numerical FAIR scores. The parser identifies each sub-score through regular-expression pattern matching that targets the expected table structure, while a second pass detects the total FAIR score when reported. This ensures that the entire scoring process is deterministic, reproducible, and independent of LLM chain-of-thought variability.

Handling Formatting Variability and Edge Cases

Because LLM-generated markdown may occasionally contain commentary, missing table rows, or deviations from the expected structure, we designed a fallback routine to maintain robustness. Outputs that fail pattern matching—such as missing FAIR components, malformed rows, or chain-of-thought text inside tables—are automatically logged, assigned default placeholder values (0 or NA), and flagged for review. The parser also includes internal consistency checks; scores exceeding allowable thresholds (e.g., F > 17) trigger invalidation of the entire output and result in exclusion from downstream analysis.

Quality Control and Rate-Limiting Procedures

To prevent API overload and ensure uniform evaluation, a fixed delay between model calls was implemented. Outputs that exceeded maximum thresholds or failed internal consistency checks were automatically flagged and re-evaluated when appropriate. This provides an additional safeguard against occasional LLM hallucinations or formatting inconsistencies.

Reproducibility and Open-Source Availability

The full workflow—including the LLM prompting templates, rule-based parsing logic, error-handling routines, scoring pipeline, and supporting scripts—is available for reproduction and audit in the project’s public GitHub repository.

GitHub repository:
[https://github.com/JialinDong/LLM-FAIR-PromptEngineering](https://github.com/JialinDong/LLM-FAIR-PromptEngineering?utm_source=chatgpt.com)

## **Text S2.** Relationship Between PFAS Databases and Research Publications

Method

To evaluate the relationship between publicly available PFAS datasets and the corresponding volume of scientific publications across U.S. states, we implemented a keyword-based web scraping approach using Google Scholar. Search queries were systematically constructed in the format: "PFAS + [state name] + [environmental matrix]", where the environmental matrices were one of four categories: groundwater, surface water, drinking water, or soil. This process was repeated for all 50 U.S. states and across all four environmental media, resulting in a comprehensive matrix of publication counts. Automated searches were conducted using a Python script that leveraged the scholarly and requests libraries to extract the number of search results (i.e., article titles) returned by Google Scholar for each query. To minimize bias and avoid citation inflation, only the total number of search hits was recorded—citation metrics were excluded. Data extraction was performed in early 2024 to maintain consistency in the temporal scope. The resulting data were aggregated and visualized using Python’s pandas and matplotlib libraries, with each environmental medium plotted as a separate bar for each state. In addition to visualization, statistical analyses were conducted to assess patterns and identify potential gaps between publicly accessible PFAS datasets and corresponding research activity.

Result

Citation activity is concentrated in the West Coast (California, Washington), Northeast (New York, New Jersey), and Midwest (Michigan, Ohio), with Washington, New York, and California leading across all matrices. In contrast, the Mountain West and central Plains states, such as Wyoming, Montana, and Nebraska, show low citation activity, reflecting a broader trend where research efforts are focused in populous, industrialized regions while large central areas remain under-monitored.

The relationship between PFAS databases, evaluation metrics (such as FAIR scores), and research applications is complex. State-level analysis reveals no strong correlation between higher FAIR scores, data quality, or integrability and increased citation activity. While the number of data sources shows a weak positive correlation with citation count (r = 0.15), all other metrics—including FAIR-Score (r = -0.27), Interoperability (r = -0.30), and Data Quality (r = -0.11)—exhibit weak negative correlations. These findings indicate that states with more standardized or higher-quality PFAS datasets are not necessarily those receiving greater research attention. Instead, citation trends appear to be driven more by external factors such as regional contamination concerns, policy priorities, and the presence of active research institutions, rather than by the technical robustness or FAIR compliance of the datasets themselves.

## **Figure S1.** The number of current published PFAS relevant scientific papers for each state

## **Table S2.** Model-Specific Robustness in Markdown Output Generation and Parsing

| Model | Total Evaluations | Valid Outputs | Invalid Outputs | Failure Rate (%) | Notes |
| --- | --- | --- | --- | --- | --- |
| gpt-4o | 200 | 197 | 3 | 1.5% | Minor formatting deviations |
| gpt-4o-mini | 32 | 32 | 0 | 0% | Fully stable across tests |
| gpt-4-turbo | 33 | 32 | 1 | 3.0% | One missing delimiter |
| gpt-3.5-turbo | 33 | 32 | 1 | 3.0% | Occasional extraneous text |
| gpt-4 | 32 | 32 | 0 | 0% | Fully stable across tests |

## **Table S3.** Summary of FAIR Scores for PFAS Data Across Different Environmental Matrices

| Environmental Matrix | Number of databases included | FAIR Total (%) | F-Score  (%) | A-Score (%) | I-Score  (%) | R-Score  (%) |
| --- | --- | --- | --- | --- | --- | --- |
| Groundwater | 39 | 45.18% | 39.22% | 68.97% | 41.03% | 31.50% |
| Surface Water | 26 | 53.57% | 46.82% | 78.40% | 48.50% | 40.57% |
| Drinking Water | 31 | 52.57% | 44.21% | 77.74% | 43.15% | 45.16% |
| Soil | 14 | 49.59% | 43.70% | 75.71% | 40.18% | 38.78% |

## **Table S4.** PFAS databases FAIR scores results evaluated by few-shot CoT LLM (ChatGPT 4o)

| Class | States | Data Source | FAIR-Score | F-Score (%) | A-Score (%) | I-Score (%) | R-Score (%) |
| --- | --- | --- | --- | --- | --- | --- | --- |
| Groundwater | Multi-state | EWG | 37.169 | 41.176 | 70 | 37.5 | 0 |
| Groundwater | Multi-state | UCMR3 | 72.967 | 52.941 | 80 | 87.5 | 71.429 |
| Groundwater | Multi-state | PPL | 69.842 | 52.941 | 80 | 75 | 71.429 |
| Groundwater | Multi-state | UCMR5 | 69.842 | 52.941 | 80 | 75 | 71.429 |
| Groundwater | Single-state | Alaska DEC | 37.169 | 41.176 | 70 | 37.5 | 0 |
| Groundwater | Single-state | GAMA | 69.842 | 52.941 | 80 | 75 | 71.429 |
| Groundwater | Multi-state | USGS | 69.842 | 52.941 | 80 | 75 | 71.429 |
| Groundwater | Single-state | NMED | 37.169 | 41.176 | 70 | 37.5 | 0 |
| Groundwater | Single-state | ADEQ | 62.122 | 47.059 | 80 | 50 | 71.429 |
| Groundwater | Single-state | CDPHE | 40.299 | 29.412 | 80 | 37.5 | 14.286 |
| Groundwater | Single-state | (Jackson Hole airport) | 31.103 | 29.412 | 70 | 25 | 0 |
| Groundwater | Single-state | EGLE | 71.313 | 58.824 | 80 | 75 | 71.429 |
| Groundwater | Single-state | MPCA | 42.169 | 41.176 | 90 | 37.5 | 0 |
| Groundwater | Single-state | Utah DEQ | 68.372 | 47.059 | 80 | 75 | 71.429 |
| Groundwater | Single-state | North Carolina DEQ | 69.842 | 52.941 | 80 | 75 | 71.429 |
| Groundwater | Single-state | IDEQ | 24.044 | 41.176 | 30 | 25 | 0 |
| Groundwater | Single-state | Seattle Public Utilities | 22.978 | 29.412 | 50 | 12.5 | 0 |
| Groundwater | Single-state | NDDEQ | 43.241 | 41.176 | 80 | 37.5 | 14.286 |
| Groundwater | Single-state | Missouri PFAS Viewer | 28.419 | 41.176 | 60 | 12.5 | 0 |
| Groundwater | Single-state | fight4zero | 27.978 | 29.412 | 70 | 12.5 | 0 |
| Groundwater | Single-state | ERIC | 69.842 | 52.941 | 80 | 75 | 71.429 |
| Groundwater | Single-state | EPD | 32.978 | 29.412 | 90 | 12.5 | 0 |
| Groundwater | Single-state | ERISIllinois EPA's PFAS Sampling Network | 39.044 | 41.176 | 90 | 25 | 0 |
| Groundwater | Single-state | DNR | 72.342 | 52.941 | 90 | 75 | 71.429 |
| Groundwater | Single-state | PFAS in NYS | 22.353 | 29.412 | 60 | 0 | 0 |
| Groundwater | Single-state | Arkansas Department of Energy and Environment | 34.044 | 41.176 | 70 | 25 | 0 |
| Groundwater | Single-state | ADEM | 62.122 | 47.059 | 80 | 50 | 71.429 |
| Groundwater | Single-state | Ohio Environmental Protection Agency | 63.592 | 52.941 | 80 | 50 | 71.429 |
| Groundwater | Single-state | Virginia Department of Health Office of Drinking Water | 62.122 | 47.059 | 80 | 50 | 71.429 |
| Groundwater | Single-state | TDEC | 0 | 0 | 0 | 0 | 0 |
| Groundwater | Single-state | Evaluation of Kentucky Community Drinking Water | 0 | 0 | 0 | 0 | 0 |
| Groundwater | Single-state | Maine.gov | 60.651 | 41.176 | 80 | 50 | 71.429 |
| Groundwater | Single-state | Ambient Surface Water PFAS Project | 34.669 | 41.176 | 60 | 37.5 | 0 |
| Groundwater | Single-state | GOV | 54.139 | 29.412 | 80 | 50 | 57.143 |
| Groundwater | Single-state | MDE | 37.169 | 41.176 | 70 | 37.5 | 0 |
| Groundwater | Single-state | Safe Waters | 31.103 | 29.412 | 70 | 25 | 0 |
| Groundwater | Single-state | NHDES | 62.122 | 47.059 | 80 | 50 | 71.429 |
| Groundwater | Single-state | NJDEP | 0 | 0 | 0 | 0 | 0 |
| Groundwater | Single-state | Massachusetts Sierra Club | 27.978 | 29.412 | 70 | 12.5 | 0 |
| Surface Water | Multi-state | UCMR5 | 62.122 | 47.059 | 80 | 50 | 71.429 |
| Surface Water | Multi-state | PPL | 57.71 | 29.412 | 80 | 50 | 71.429 |
| Surface Water | Multi-state | UCMR3,5 | 72.967 | 52.941 | 80 | 87.5 | 71.429 |
| Surface Water | Single-state | DSHS | 36.544 | 41.176 | 80 | 25 | 0 |
| Surface Water | Single-state | SFEI | 62.747 | 47.059 | 70 | 62.5 | 71.429 |
| Surface Water | Single-state | CDPHE | 39.044 | 41.176 | 90 | 25 | 0 |
| Surface Water | Single-state | DCD | 72.416 | 88.235 | 80 | 50 | 71.429 |
| Surface Water | Single-state | University of Colorado at Colorado Springs | 72.416 | 88.235 | 80 | 50 | 71.429 |
| Surface Water | Single-state | EGLE | 74.842 | 52.941 | 100 | 75 | 71.429 |
| Surface Water | Single-state | UCMR3.UCMR5 | 69.842 | 52.941 | 80 | 75 | 71.429 |
| Surface Water | Single-state | NDDEQ | 41.14 | 47.059 | 80 | 37.5 | 0 |
| Surface Water | Single-state | Missouri PFAS Viewer | 22.978 | 29.412 | 50 | 12.5 | 0 |
| Surface Water | Multi-state | DEQ | 42.169 | 41.176 | 90 | 37.5 | 0 |
| Surface Water | Multi-state | Florida Department of Environmental Protection | 37.799 | 29.412 | 70 | 37.5 | 14.286 |
| Surface Water | Single-state | EPD | 57.08 | 41.176 | 80 | 50 | 57.143 |
| Surface Water | Single-state | Illinois EPA's PFAS Sampling Network | 39.669 | 41.176 | 80 | 37.5 | 0 |
| Surface Water | Single-state | UCMR5 DEQ UCMR3 | 69.842 | 52.941 | 80 | 75 | 71.429 |
| Surface Water | Single-state | WRRC | 34.044 | 41.176 | 70 | 25 | 0 |
| Surface Water | Single-state | Virginia Department of Health Office of Drinking Water | 43.866 | 41.176 | 70 | 50 | 14.286 |
| Surface Water | Single-state | TDEC | 48.913 | 35.294 | 80 | 37.5 | 42.857 |
| Surface Water | Single-state | Evaluation of Kentucky Community Drinking Water | 34.228 | 29.412 | 70 | 37.5 | 0 |
| Surface Water | Single-state | Maine.gov | 46.812 | 41.176 | 80 | 37.5 | 28.571 |
| Surface Water | Single-state | PFAS Drinking Water Sampling Results | 63.592 | 52.941 | 80 | 50 | 71.429 |
| Surface Water | Single-state | MDE | 62.122 | 47.059 | 80 | 50 | 71.429 |
| Surface Water | Single-state | USGS | 74.438 | 58.824 | 80 | 87.5 | 71.429 |
| Drinking Water | Single-state | Alaska DEC | 53.955 | 41.176 | 80 | 37.5 | 57.143 |
| Drinking Water | Multi-state | DoD | 52.085 | 29.412 | 70 | 37.5 | 71.429 |
| Drinking Water | Multi-state | EWG | 37.169 | 41.176 | 70 | 37.5 | 0 |
| Drinking Water | Multi-state | PPL | 69.842 | 52.941 | 80 | 75 | 71.429 |
| Drinking Water | Single-state | CA.gov | 62.122 | 47.059 | 80 | 50 | 71.429 |
| Drinking Water | Single-state | GAMA | 63.592 | 52.941 | 80 | 50 | 71.429 |
| Drinking Water | Single-state | gispublic | 64.622 | 47.059 | 90 | 50 | 71.429 |
| Drinking Water | Single-state | NMED | 62.122 | 47.059 | 80 | 50 | 71.429 |
| Drinking Water | Single-state | CDPHE | 39.044 | 41.176 | 90 | 25 | 0 |
| Drinking Water | Single-state | OHA | 62.122 | 47.059 | 80 | 50 | 71.429 |
| Drinking Water | Single-state | DOH | 68.372 | 47.059 | 80 | 75 | 71.429 |
| Drinking Water | Single-state | NDDEQ | 37.169 | 41.176 | 70 | 37.5 | 0 |
| Drinking Water | Single-state | fight4zero | 24.853 | 29.412 | 70 | 0 | 0 |
| Drinking Water | Single-state | Wisconsin Department of Natural Resource | 34.044 | 41.176 | 70 | 25 | 0 |
| Drinking Water | Single-state | Village of East Troy | 27.978 | 29.412 | 70 | 12.5 | 0 |
| Drinking Water | Single-state | EPD | 34.044 | 41.176 | 70 | 25 | 0 |
| Drinking Water | Single-state | Illinois EPA | 69.842 | 52.941 | 80 | 75 | 71.429 |
| Drinking Water | Single-state | PFAS IN NYS | 51.675 | 47.059 | 90 | 12.5 | 57.143 |
| Drinking Water | Single-state | DEP | 37.169 | 41.176 | 70 | 37.5 | 0 |
| Drinking Water | Single-state | VIRGINIA DEPARTMENT OF HEALTH | 60.651 | 41.176 | 80 | 50 | 71.429 |
| Drinking Water | Single-state | TDEC | 62.122 | 47.059 | 80 | 50 | 71.429 |
| Drinking Water | Single-state | Indiana Department of Environmental Management | 37.169 | 41.176 | 70 | 37.5 | 0 |
| Drinking Water | Single-state | Maine DWP | 62.122 | 47.059 | 80 | 50 | 71.429 |
| Drinking Water | Single-state | PPL PFAS Drinking Water Sampling Results | 63.592 | 52.941 | 80 | 50 | 71.429 |
| Drinking Water | Single-state | MDE | 63.592 | 52.941 | 80 | 50 | 71.429 |
| Drinking Water | Single-state | DOH report | 34.044 | 41.176 | 70 | 25 | 0 |
| Drinking Water | Single-state | MassGOV | 68.372 | 47.059 | 80 | 75 | 71.429 |
| Drinking Water | Single-state | usgs | 63.592 | 52.941 | 80 | 50 | 71.429 |
| Drinking Water | Single-state | Commenwealth of Mass | 36.544 | 41.176 | 80 | 25 | 0 |
| Drinking Water | Single-state | DWGPD | 65.247 | 47.059 | 80 | 62.5 | 71.429 |
| Drinking Water | Single-state | State of Rhode Island Department of Health | 60.651 | 41.176 | 80 | 50 | 71.429 |
| Soil | Multi-state | PPL | 24.853 | 29.412 | 70 | 0 | 0 |
| Soil | Single-state | DSHS | 36.544 | 41.176 | 80 | 25 | 0 |
| Soil | Single-state | PFAS Project Lab | 45.294 | 41.176 | 90 | 50 | 0 |
| Soil | Single-state | NMED(email) | 62.122 | 47.059 | 80 | 50 | 71.429 |
| Soil | Single-state | RWRD | 58.151 | 41.176 | 70 | 50 | 71.429 |
| Soil | Single-state | CDPHE | 52.038 | 35.294 | 80 | 50 | 42.857 |
| Soil | Single-state | University of Colorado at Colorado Springs | 72.416 | 88.235 | 80 | 50 | 71.429 |
| Soil | Single-state | fight4zero | 31.103 | 29.412 | 70 | 25 | 0 |
| Soil | Single-state | EWG | 19.853 | 29.412 | 50 | 0 | 0 |
| Soil | Single-state | Maine.gov/ PPL | 62.122 | 47.059 | 80 | 50 | 71.429 |
| Soil | Single-state | DOH report | 34.228 | 29.412 | 70 | 37.5 | 0 |
| Soil | Single-state | UNIVERSITY OF VERMONT | 62.122 | 47.059 | 80 | 50 | 71.429 |
| Soil | Single-state | USGS | 69.842 | 52.941 | 80 | 75 | 71.429 |
| Soil | Single-state | EPA | 63.592 | 52.941 | 80 | 50 | 71.429 |

## **Table S5.** PFAS databases FAIR scores statistical analysis results evaluated by few-shot CoT LLM (ChatGPT 4o)

| Class | Drinking Water | Groundwater | Soil | Surface Water |
| --- | --- | --- | --- | --- |
| FAIR-Score_count | 31.00 | 39.00 | 14.00 | 25.00 |
| FAIR-Score_mean | 52.57 | 45.18 | 49.59 | 53.57 |
| FAIR-Score_std | 14.46 | 21.62 | 17.41 | 15.74 |
| FAIR-Score_min | 24.85 | 0.00 | 19.85 | 22.98 |
| FAIR-Score_25% | 37.17 | 31.10 | 34.81 | 39.67 |
| FAIR-Score_50% | 60.65 | 40.30 | 55.09 | 57.08 |
| FAIR-Score_75% | 63.59 | 65.98 | 62.12 | 69.84 |
| FAIR-Score_max | 69.84 | 72.97 | 72.42 | 74.84 |
| F-Score (%)_count | 31.00 | 39.00 | 14.00 | 25.00 |
| F-Score (%)_mean | 44.21 | 39.22 | 43.70 | 46.82 |
| F-Score (%)_std | 6.60 | 14.43 | 15.43 | 14.95 |
| F-Score (%)_min | 29.41 | 0.00 | 29.41 | 29.41 |
| F-Score (%)_25% | 41.18 | 29.41 | 30.88 | 41.18 |
| F-Score (%)_50% | 47.06 | 41.18 | 41.18 | 41.18 |
| F-Score (%)_75% | 47.06 | 50.00 | 47.06 | 52.94 |
| F-Score (%)_max | 52.94 | 58.82 | 88.24 | 88.24 |
| A-Score (%)_count | 31.00 | 39.00 | 14.00 | 25.00 |
| A-Score (%)_mean | 77.74 | 68.97 | 75.71 | 78.40 |
| A-Score (%)_std | 6.17 | 23.15 | 9.38 | 8.98 |
| A-Score (%)_min | 70.00 | 0.00 | 50.00 | 50.00 |
| A-Score (%)_25% | 70.00 | 70.00 | 70.00 | 80.00 |
| A-Score (%)_50% | 80.00 | 80.00 | 80.00 | 80.00 |
| A-Score (%)_75% | 80.00 | 80.00 | 80.00 | 80.00 |
| A-Score (%)_max | 90.00 | 90.00 | 90.00 | 100.00 |
| I-Score (%)_count | 31.00 | 39.00 | 14.00 | 25.00 |
| I-Score (%)_mean | 43.15 | 41.03 | 40.18 | 48.50 |
| I-Score (%)_std | 18.77 | 25.80 | 20.90 | 19.54 |
| I-Score (%)_min | 0.00 | 0.00 | 0.00 | 12.50 |
| I-Score (%)_25% | 31.25 | 25.00 | 28.13 | 37.50 |
| I-Score (%)_50% | 50.00 | 37.50 | 50.00 | 50.00 |
| I-Score (%)_75% | 50.00 | 62.50 | 50.00 | 50.00 |
| I-Score (%)_max | 75.00 | 87.50 | 75.00 | 87.50 |
| R-Score (%)_count | 31.00 | 39.00 | 14.00 | 25.00 |
| R-Score (%)_mean | 45.16 | 31.50 | 38.78 | 40.57 |
| R-Score (%)_std | 34.23 | 35.02 | 35.63 | 33.17 |
| R-Score (%)_min | 0.00 | 0.00 | 0.00 | 0.00 |
| R-Score (%)_25% | 0.00 | 0.00 | 0.00 | 0.00 |
| R-Score (%)_50% | 71.43 | 0.00 | 57.14 | 57.14 |
| R-Score (%)_75% | 71.43 | 71.43 | 71.43 | 71.43 |
| R-Score (%)_max | 71.43 | 71.43 | 71.43 | 71.43 |

## **Figure S2.** Distribution of FAIR Principles Scores by State.

## **Figure S3.** F vs A Scores by different database (FAIR Size Weighted)

## **Figure S4.** I vs R Scores by different database (FAIR Size Weighted)

## **Text S3.** Groundwater PFOA Concentration Data Distribution Summary

The groundwater dataset contains 149,293 samples collected nationwide, of which 108,639 (≈73%) included valid PFOA concentration measurements. Approximately 40,654 samples (27%) lacked quantitative data. Samples were classified into four source categories: non-PFAS-contaminated (n = 97,476; 65%), mixed sources (n = 41,688; 28%), PFAS-contaminated (n = 5,545; 4%), and unknown (n = 307; <1%).

Measured PFOA concentrations displayed extreme variability, ranging from non-detects to a maximum of 6,570,000 ng/L. The distribution was heavily right-skewed, with the majority of samples at or near detection limits but a minority of sites exhibiting extraordinarily high contamination. The median concentration was 0 ng/L, with the 25th and 75th percentiles at 0 and 5.57 ng/L, respectively. The 90th and 95th percentiles rose to 21.5 ng/L and 130 ng/L, respectively. The mean concentration (405 ng/L) was strongly influenced by a small number of extreme outliers, underscoring the importance of localized contamination hotspots in shaping the overall distribution.

Geographically, sample coverage was dominated by a few states. Maine (n = 40,279), California (n = 31,164), and New Jersey (n = 30,983) accounted for more than two-thirds of all groundwater samples, reflecting intensive regional monitoring efforts. Other states contributing substantial data included Ohio (n = 3,868), Texas (n = 3,553), and Michigan (n = 2,711), along with Florida, Arizona, New York, and Massachusetts. This uneven distribution highlights the heterogeneity of groundwater monitoring, with some states undertaking large-scale testing while others remain underrepresented.

## **Figure S5.** Groundwater PFOA Log Concentration by state from non PFAS contaminated sources

## **Figure S6.** Groundwater PFOA Log Concentration by state from known PFAS contaminated sources

## **Table S6.** Groundwater PFOA trend analysis for each state based on monthly medians of PFOA concentrations from non-PFAS-contaminated sources

| state | n_months | ols_slope_per_month | ols_p | ols_direction | mk_tau | mk_p | theil_sen_slope_per_m | mk_direction | agreement | both_agree_and_significant |
| --- | --- | --- | --- | --- | --- | --- | --- | --- | --- | --- |
| AK | 32 | 0 | 1 | no change | 0 | 1 | 0 | no change | yes | FALSE |
| AL | 53 | 0 | 1 | no change | 0 | 1 | 0 | no change | yes | FALSE |
| AR | 42 | 0 | 1 | no change | 0 | 1 | 0 | no change | yes | FALSE |
| AS | 5 |  |  | insufficient data |  |  |  | insufficient data | no (insufficient data) | FALSE |
| AZ | 54 | 0 | 1 | no change | 0 | 1 | 0 | no change | yes | FALSE |
| CA | 125 | -0 | 0.9344334138198178 | no change | 0.12141935483870968 | 0.037180879634609774 | 0 | increasing | no | FALSE |
| CO | 47 | -0.31 | 0.018 | decreasing | -0.12 | 0.018253162297416838 | 0 | decreasing | yes | TRUE |
| CT | 46 | 0.049401788467468385 | 0.017 | increasing | 0.157 | 0.008 | 0 | increasing | yes | TRUE |
| DE | 37 | -0.17 | 0.176 | no change | -0.03 | 0.743 | 0 | no change | yes | FALSE |
| FL | 57 | 0 | 1 | no change | 0 | 1 | 0 | no change | yes | FALSE |
| GA | 54 | 0 | 1 | no change | 0 | 1 | 0 | no change | yes | FALSE |
| GU | 14 |  |  | insufficient data |  |  |  | insufficient data | no (insufficient data) | FALSE |
| HI | 40 | 0 | 1 | no change | 0 | 1 | 0 | no change | yes | FALSE |
| IA | 51 | 0 | 1 | no change | 0 | 1 | 0 | no change | yes | FALSE |
| ID | 45 | 0 | 1 | no change | 0 | 1 | 0 | no change | yes | FALSE |
| IL | 54 | 0 | 1 | no change | 0 | 1 | 0 | no change | yes | FALSE |
| IN | 55 | 0 | 1 | no change | 0 | 1 | 0 | no change | yes | FALSE |
| KS | 48 | 0 | 1 | no change | 0 | 1 | 0 | no change | yes | FALSE |
| KY | 38 | 0.008 | 0.4090736785510527 | no change | 0.036 | 0.43301322228963335 | 0 | no change | yes | FALSE |
| LA | 52 | 0 | 1 | no change | 0 | 1 | 0 | no change | yes | FALSE |
| MA | 62 | 0.083 | 0.196 | no change | 0.07 | 0.11681509927261649 | 0 | no change | yes | FALSE |
| MD | 61 | 0.018 | 0.20381209809175324 | no change | 0.079 | 0.06 | 0 | no change | yes | FALSE |
| ME | 29 | 0 | 1 | no change | 0 | 1 | 0 | no change | yes | FALSE |
| MI | 101 | -1.66 | 0.5432430332296274 | no change | 0.015247524752475247 | 0.7887525672699154 | 0 | no change | yes | FALSE |
| MN | 54 | 0 | 1 | no change | 0 | 1 | 0 | no change | yes | FALSE |
| MO | 61 | 0 | 1 | no change | 0 | 1 | 0 | no change | yes | FALSE |
| MP | 7 |  |  | insufficient data |  |  |  | insufficient data | no (insufficient data) | FALSE |
| MS | 53 | 0 | 1 | no change | 0 | 1 | 0 | no change | yes | FALSE |
| MT | 30 | 0 | 1 | no change | 0 | 1 | 0 | no change | yes | FALSE |
| NC | 53 | 0 | 1 | no change | 0 | 1 | 0 | no change | yes | FALSE |
| ND | 27 | 0 | 1 | no change | 0 | 1 | 0 | no change | yes | FALSE |
| NE | 46 | 0 | 1 | no change | 0 | 1 | 0 | no change | yes | FALSE |
| NH | 36 | -0.24 | 0.19844380961099192 | no change | -0.04 | 0.4979696350937686 | 0 | no change | yes | FALSE |
| NJ | 106 | 0.009 | 0.028911867552494936 | increasing | 0.10242587601078167 | 0.015 | 0 | increasing | yes | TRUE |
| NM | 48 | 0 | 1 | no change | 0 | 1 | 0 | no change | yes | FALSE |
| NN | 19 | 0 | 1 | no change | 0 | 1 | 0 | no change | yes | FALSE |
| NV | 35 | 0 | 1 | no change | 0 | 1 | 0 | no change | yes | FALSE |
| NY | 55 | 0 | 1 | no change | 0 | 1 | 0 | no change | yes | FALSE |
| OH | 53 | 0 | 1 | no change | 0 | 1 | 0 | no change | yes | FALSE |
| OK | 50 | -0 | 0.8645395636327775 | no change | -0 | 0.8897727346992708 | 0 | no change | yes | FALSE |
| OR | 47 | 0 | 1 | no change | 0 | 1 | 0 | no change | yes | FALSE |
| PA | 54 | 0 | 1 | no change | 0 | 1 | 0 | no change | yes | FALSE |
| PR | 38 | 0 | 1 | no change | 0 | 1 | 0 | no change | yes | FALSE |
| RI | 23 | -0.23 | 0.6725501345198039 | no change | 0.19367588932806323 | 0.11751027168579462 | 0 | no change | yes | FALSE |
| SC | 45 | 0 | 1 | no change | 0 | 1 | 0 | no change | yes | FALSE |
| SD | 33 | 0 | 1 | no change | 0 | 1 | 0 | no change | yes | FALSE |
| TN | 50 | 0 | 1 | no change | 0 | 1 | 0 | no change | yes | FALSE |
| TX | 58 | 0 | 1 | no change | 0 | 1 | 0 | no change | yes | FALSE |
| UT | 59 | 0 | 1 | no change | 0 | 1 | 0 | no change | yes | FALSE |
| VA | 46 | 0 | 1 | no change | 0 | 1 | 0 | no change | yes | FALSE |
| VI | 2 |  |  | insufficient data |  |  |  | insufficient data | no (insufficient data) | FALSE |
| VT | 27 | 0 | 1 | no change | 0 | 1 | 0 | no change | yes | FALSE |
| WA | 56 | 0 | 1 | no change | 0 | 1 | 0 | no change | yes | FALSE |
| WI | 54 | 0 | 1 | no change | 0 | 1 | 0 | no change | yes | FALSE |
| WV | 56 | 0.055 | 0.7704691009877287 | no change | 0.086 | 0.20589627024435334 | 0 | no change | yes | FALSE |
| WY | 26 | 0 | 1 | no change | 0 | 1 | 0 | no change | yes | FALSE |

## **Figure S7.** Surface water PFOA Concentration (ng/L) distribution by data source (log-scaled). The vertical blue line separates single-state (left) and multi-state (right) data sources. Sources and sample counts (years): Florida Department of Environmental Protection (DEP) (35, 2020–2022), Georgia Environmental Protection Division (EPD) (37, 2016), EPA Unregulated Contaminant Monitoring Rule 3 (UCMR3) (2269, 2013–2016), South Carolina Department of Health and Environmental Control (DHEC) (91, 2017–2020), University of Colorado (8, 2018–2019), Evaluation of Kentucky Community Drinking Water (80, 2019), Virginia Department of Health (DOH) Office of Drinking Water (11 locations, 2022), Texas Department of State Health Services (DSHS) (7, 2021), MDE (3, 2021), Maine.gov (70 locations, 2013–2023), EPA Unregulated Contaminant Monitoring Rule 5 (UCMR5) (2147 locations, 2023–2024).

## **Text S4.** Surface water PFOA Concentration Data Distribution Summary

The surface water dataset comprises 20,319 samples collected across the United States, of which 1,934 (≈9.5%) included valid PFOA concentration measurements. The remaining 18,385 samples lacked quantitative values. Samples were categorized into three source classifications: non-PFAS-contaminated (n = 19,194; 94%), mixed sources (n = 747; 4%), and PFAS-contaminated (n = 378; 2%).

Measured PFOA concentrations spanned a broad range from non-detects to a maximum of 11,300 ng/L. The distribution was highly skewed, with most observations near or below detection limits. The median concentration was 0.022 ng/L, with the 25th and 75th percentiles at 0.0044 and 6.2 ng/L, respectively. The 90th and 95th percentiles reached 71 ng/L and 181 ng/L, respectively. Although the mean concentration (84 ng/L) was elevated, this value was disproportionately influenced by sporadic high outliers. These findings underscore that while the majority of surface water samples contained negligible PFOA levels, a small fraction of sites exhibited extreme contamination.

Geographically, sampling was widespread, with the highest number of observations reported from Texas (n = 2,509), California (n = 1,331), Pennsylvania (n = 1,088), Illinois (n = 1,051), and Kentucky (n = 1,050). Other states, including New York (n = 876), North Carolina (n = 873), Tennessee (n = 860), Michigan (n = 822), and Maine (n = 745), also contributed substantial datasets. This broad coverage highlights the extensive monitoring of surface waters, though the high proportion of non-detects indicates limited but spatially concentrated contamination events.

## **Figure S8.** Surface water PFOA Log Concentration by state including all PFAS sources

## **Figure S9.** Surface water PFOA Log Concentration by state from non PFAS contaminated source

## **Table S7.** Surface water PFOA trend analysis for each state based on monthly medians of PFOA concentrations from non-PFAS-contaminated sources

| state | n_months | ols_slope_per_month | ols_p | ols_direction | mk_tau | mk_p | theil_sen_slope_per_m | mk_direction | agreement | both_agree_and_significant |
| --- | --- | --- | --- | --- | --- | --- | --- | --- | --- | --- |
| AL | 33 | -0.0002 | 2.7725281642543332e-05 | decreasing | -0.4981 | 2.7721536721703188e-05 |  | decreasing | decreasing | TRUE |
| CA | 6 | ###### | 0.8988597966344839 | decreasing | -0.0667 | 1 |  | decreasing | decreasing | FALSE |
| CO | 2 |  |  |  |  |  |  |  | disagree | FALSE |
| CT | 5 | -0.0002 | 0.053158166434675656 | decreasing | -1 | 0.01004 |  | decreasing | decreasing | FALSE |
| DE | 10 | -0.0002 | 0.00848 | decreasing | -0.2889 | 0.2415665869689727 |  | decreasing | decreasing | FALSE |
| FL | 6 | 0.00089 | 0.9060826069021959 | increasing | 0.3333333333333333 | 0.3901541483019262 |  | increasing | increasing | FALSE |
| GA | 15 | -0.0003 | 3.7416506175866974e-06 | decreasing | -0.219 | 0.24734302362110716 |  | decreasing | decreasing | FALSE |
| IA | 3 | -0.0005 | 0.16566519637105814 | decreasing | -1 | 0.1904302638255242 |  | decreasing | decreasing | FALSE |
| IL | 2 |  |  |  |  |  |  |  | disagree | FALSE |
| IN | 1 |  |  |  |  |  |  |  | disagree | FALSE |
| KY | 7 | -0.0001 | 8.325521867058712e-07 | decreasing | -0.4286 | 0.17629637444051127 |  | decreasing | decreasing | FALSE |
| LA | 1 |  |  |  |  |  |  |  | disagree | FALSE |
| MA | 11 | 1.679931418608204e-05 | 0.8661403670714982 | increasing | 0.21818181818181817 | 0.35349546432760115 |  | increasing | increasing | FALSE |
| MD | 1 |  |  |  |  |  |  |  | disagree | FALSE |
| ME | 3 | 0.00011 | 0.65741 | increasing | 0.3333333333333333 | 1 |  | increasing | increasing | FALSE |
| MI | 1 |  |  |  |  |  |  |  | disagree | FALSE |
| NC | 20 | -0.0002 | 3.384828201103315e-10 | decreasing | -0.2 | 0.20852417225230457 |  | decreasing | decreasing | FALSE |
| NH | 10 | 0.00012 | 0.04801 | increasing | 0.35555555555555557 | 0.14323490752466972 |  | increasing | increasing | FALSE |
| NJ | 25 | -0.0002 | 7.823983219220647e-10 | decreasing | -0.44 | 0.00149 |  | decreasing | decreasing | TRUE |
| NY | 16 | -0.0002 | 1.1488192315345036e-12 | decreasing | -0.4167 | 0.019492109652483824 |  | decreasing | decreasing | TRUE |
| OH | 9 | -0.0002 | 2.430884006803213e-08 | decreasing | -0.4444 | 0.08532 |  | decreasing | decreasing | FALSE |
| PA | 9 | -0.0005 | 0.2176787361561125 | decreasing | 0.05556 | 0.9086766781799485 |  | increasing | disagree | FALSE |
| RI | 1 |  |  |  |  |  |  |  | disagree | FALSE |
| SC | 31 | -0.0572 | 3.823285275220627e-05 | decreasing | -0.5204 | 2.3982133676492268e-05 |  | decreasing | decreasing | TRUE |
| TN | 13 | -0.0001 | 8.916472424377183e-06 | decreasing | 0.02564 | 0.9478777812782535 |  | increasing | disagree | FALSE |
| TX | 15 | -0.0109 | 0.7122499088167309 | decreasing | -0.1619 | 0.4001637767637314 |  | decreasing | decreasing | FALSE |
| VA | 6 | 0.00032 | 0.010209407773531508 | increasing | 0.3333333333333333 | 0.3901541483019262 |  | increasing | increasing | FALSE |
| WV | 4 | 0.00018 | 0.8853553289014598 | increasing | -0.3333 | 0.6830913983096087 |  | decreasing | disagree | FALSE |

## **Text S5.** Drinking water PFOA Concentration Data Distribution Summary

The compiled dataset comprises 71,518 drinking water samples collected across the United States, with 71,449 (99.9%) measurements of PFOA concentrations available. Most entries include geographic coordinates (≈81%) and sampling location information (≈59%), ensuring broad spatial coverage. Samples were derived from three categories of source classification: non-PFAS-contaminated (51,996; 73%), PFAS-contaminated (14,669; 21%), and mixed sources (4,853; 7%). Nearly all entries were labeled as drinking water samples, confirming the dataset’s focus on human exposure pathways.

PFOA concentrations exhibit a highly skewed distribution, with the majority of samples at or near detection limits but punctuated by a small proportion of high values. The median concentration was 0.63 ng/L, with the 75th and 95th percentiles at 6.34 ng/L and 34.8 ng/L, respectively. Although the mean concentration was 10.6 ng/L, this value is strongly influenced by extreme outliers, with maximum concentrations reaching 8,800 ng/L. These results indicate that while most drinking water sources show low or non-detectable levels, localized sites of severe contamination persist.

Geographically, sample density is uneven. California (n = 24,321) and Massachusetts (n = 19,980) contributed the largest number of samples, followed by Texas (n = 6,282), Washington (n = 4,625), and Wisconsin (n = 3,475). Several states such as Vermont (n = 2,071), Maine (n = 1,306), and Colorado (n = 1,222) provided smaller but regionally important datasets. This spatial distribution underscores the heterogeneity in both monitoring intensity and contamination levels across the country.

## **Figure S10.** Drinking water PFOA Log Concentration by state from non PFAS contaminated source

## **Figure S11.** Drinking water PFOA Log Concentration by state from PFAS contaminated source

## **Figure S12.** Drinking Water PFOA Concentration Distribution by State (All data). The horizontal blue dashed line represents a concentration of 4 ng/L.

## **Table S8.** Drinking water PFOA trend analysis for each state based on monthly medians of PFOA concentrations from all sources data

| state | n_months | ols_slope_per_month | ols_p | ols_direction | mk_tau | mk_p | theil_sen_slope_per_m | mk_direction | agreement | both_agree_and_significant |
| --- | --- | --- | --- | --- | --- | --- | --- | --- | --- | --- |
| Massachusetts | 97 | -0.14008 | 1.0426336573517698e-10 | decreasing | -0.33376 | 1.2761132857086466e-06 | -0.07417 | decreasing | TRUE | TRUE |
| Michigan | 19 | -2.39202 | 0.011204956192788712 | decreasing | -0.4269 | 0.0034 | -0.09864 | decreasing | TRUE | TRUE |
| Kansas | 8 | -1.53988 | 0.00057 | decreasing | -0.78571 | 0.00937 | -1.57643 | decreasing | TRUE | TRUE |
| Wisconsin | 29 | 0.061773399014778324 | 2.9243711514918812e-05 | increasing | 0.37438423645320196 | 0.00052 | 0 | increasing | TRUE | TRUE |
| Florida | 23 | 0.6062252964426877 | 0.10055459091162669 | stable | 0.6403162055335968 | 2.1039176635540002e-05 | 0.48571428571428577 | increasing | FALSE | FALSE |
| Georgia | 1 |  |  | insufficient_data |  |  |  | insufficient_data | FALSE | FALSE |
| Hawaii | 2 |  |  | insufficient_data |  |  |  | insufficient_data | FALSE | FALSE |
| Mississippi | 1 |  |  | insufficient_data |  |  |  | insufficient_data | FALSE | FALSE |
| Nebraska | 1 |  |  | insufficient_data |  |  |  | insufficient_data | FALSE | FALSE |
| Puerto Rico | 1 |  |  | insufficient_data |  |  |  | insufficient_data | FALSE | FALSE |
| Utah | 1 |  |  | insufficient_data |  |  |  | insufficient_data | FALSE | FALSE |
| Wyoming | 1 |  |  | insufficient_data |  |  |  | insufficient_data | FALSE | FALSE |
| Montana | 9 | -0.84667 | 0.09631 | stable | -0.5 | 0.05015 | -0.26667 | stable | FALSE | FALSE |
| Pennsylvania | 43 | 0.20899652672908486 | 0.00445 | increasing | 0.2059800664451827 | 0.051236496638789264 | 0.16421052631578945 | stable | FALSE | FALSE |
| Illinois | 7 | -1.55893 | 0.5456918579668675 | stable | -0.61905 | 0.07151 | -0.38333 | stable | FALSE | FALSE |
| Arkansas | 4 | -20.765 | 0.05889 | stable | -1 | 0.08943 | -21.025 | stable | FALSE | FALSE |
| Rhode Island | 9 | -1.39792 | 0.22479948015007262 | stable | -0.47222 | 0.09349 | -0.4575 | stable | FALSE | FALSE |
| Oregon | 13 | -0.03297 | 0.11117295276905718 | stable | -0.15385 | 0.14157886335524394 | 0 | stable | FALSE | FALSE |
| Maine | 34 | -0.67019 | 0.01631 | decreasing | -0.16043 | 0.14522560763807646 | 0 | stable | FALSE | FALSE |
| New Hampshire | 33 | -0.13189 | 0.1065615316784765 | stable | -0.17235 | 0.16291488381307118 | -0.1 | stable | FALSE | FALSE |
| Maryland | 7 | -55.2225 | 0.35994810896743895 | stable | -0.47619 | 0.17156320646337742 | -0.7125 | stable | FALSE | FALSE |
| California | 89 | -0.12211 | 8.696995842629369e-05 | decreasing | -0.08606 | 0.18570959249214636 | 0 | stable | FALSE | FALSE |
| Vermont | 53 | -0.04638 | 0.054554034784085555 | stable | -0.082 | 0.26654468450567737 | 0 | stable | FALSE | FALSE |
| Oklahoma | 16 | -0.43706 | 0.35312473446227544 | stable | -0.18333 | 0.33611989055105695 | -0.14833 | stable | FALSE | FALSE |
| Washington | 30 | 0.024972191323692983 | 0.1433650901840677 | stable | 0.059770114942528735 | 0.3885555402779084 | 0 | stable | FALSE | FALSE |
| South Carolina | 23 | 0.17830039525691702 | 0.4277135206693472 | stable | 0.11857707509881422 | 0.4424 | 0.18461538461538465 | stable | FALSE | FALSE |
| Minnesota | 8 | -76.6933 | 0.07844 | stable | -0.25 | 0.45442730225683414 | -0.475 | stable | FALSE | FALSE |
| Texas | 33 | -0.04286 | 0.8122263451813203 | stable | -0.0928 | 0.45698471235102955 | -0.08547 | stable | FALSE | FALSE |
| Delaware | 10 | 0.3136363636363635 | 0.7556607619685404 | stable | 0.2 | 0.4742743507673295 | 0.49374999999999997 | stable | FALSE | FALSE |
| Virginia | 15 | -0.16731 | 0.8285202729486195 | stable | -0.11429 | 0.5134356789947918 | 0 | stable | FALSE | FALSE |
| Alaska | 12 | -0.9847 | 0.12286520761142153 | stable | -0.15152 | 0.53521 | -0.12083 | stable | FALSE | FALSE |
| North Dakota | 3 | -1.75 | 0.3333333333333332 | stable | -0.66667 | 0.54029 | -1.75 | stable | FALSE | FALSE |
| New York | 17 | -0.06275 | 0.5880127049555037 | stable | -0.07353 | 0.6152511311177378 | 0 | stable | FALSE | FALSE |
| Colorado | 30 | 0.20423804226918793 | 0.35833863967119095 | stable | 0.052873563218390804 | 0.6629351304596702 | 0 | stable | FALSE | FALSE |
| Arizona | 4 | -0.554 | 0.6534290024130234 | stable | -0.33333 | 0.7340951823194757 | -1.33 | stable | FALSE | FALSE |
| New Jersey | 9 | -0.23833 | 0.6253593390319433 | stable | -0.11111 | 0.7544541774940892 | -0.0925 | stable | FALSE | FALSE |
| North Carolina | 13 | -0.00549 | 0.9824553554717761 | stable | 0.02564 | 0.9466494029669688 | 0 | stable | FALSE | FALSE |
| Tennessee | 7 | -0.56429 | 0.6986825488454602 | stable | -0.04762 | 1 | -0.33333 | stable | FALSE | FALSE |
| Ohio | 3 | 0.38749999999999996 | 0.9060010500379626 | stable | 0.3333333333333333 | 1 | 0.38749999999999996 | stable | FALSE | FALSE |
| South Dakota | 12 | 0.1438811188811186 | 0.9397306342180607 | stable | 0.015151515151515152 | 1 | 0 | stable | FALSE | FALSE |
| Indiana | 34 | 0 | 1 | stable | 0 | 1 | 0 | stable | FALSE | FALSE |
| New Mexico | 7 | 0 | 1 | stable | 0 | 1 | 0 | stable | FALSE | FALSE |

## **Text S6.** Soil PFOA Concentration Data Distribution Summary

The soil dataset contains 2,710 samples collected across multiple U.S. states, with 2,259 (83%) providing valid PFOA concentration measurements. Approximately 451 samples lacked quantitative data. Samples were classified into four source categories: non-PFAS-contaminated (n = 1,911; 71%), PFAS-contaminated (n = 745; 27%), mixed sources (n = 19; <1%), and unknown (n = 35; 1%).

PFOA concentrations in soil span several orders of magnitude, ranging from non-detects to a maximum of 195,000. The distribution is highly right-skewed, with most values clustering at relatively low levels but punctuated by extreme contamination hotspots. The median concentration was 595, with the 25th and 75th percentiles at 51 and 2,020, respectively. The 90th and 95th percentiles reached 5,800 and 12,630, respectively. Although the mean concentration (2,704) was elevated, it was disproportionately influenced by a small number of extremely high observations. These findings highlight the heterogeneous nature of soil contamination, where most sites contain relatively modest levels but a subset exhibits severe contamination.

Geographically, the dataset is dominated by samples from Maine (n = 1,675; >60% of total), followed by Colorado (n = 276), Alaska (n = 259), Vermont (n = 195), and New Hampshire (n = 170). Other states, including California, New Jersey, Arizona, Hawaii, and Texas, contributed smaller sample sizes. This uneven distribution emphasizes the intensive monitoring efforts in select states, particularly Maine, which strongly shapes the national profile of soil contamination in this dataset.

## **Figure S13.** Soil PFOA Log Concentration by state and year from PFAS contaminated source
